# The costs of interventions for type 2 diabetes mellitus, hypertension and cardiovascular disease in South Africa – a systematic literature review

**DOI:** 10.1186/s12889-022-14730-4

**Published:** 2022-12-12

**Authors:** Sithabiso D. Masuku, Nkgomeleng Lekodeba, Gesine Meyer-Rath

**Affiliations:** 1 Health Economics and Epidemiology Research Office, Department of Internal Medicine, School of Clinical Medicine, Faculty of Health Sciences, University of the Witwatersrand, Unit 2, 39 Empire Road, Parktown, Johannesburg, 2193 South Africa; 2 Department of Global Health, Boston University, Boston, MA USA

**Keywords:** Hypertension, Diabetes, Cardiovascular disease, Cost, South Africa

## Abstract

**Background:**

In the context of a move to universal health coverage, three separate systematic reviews were conducted to summarise available evidence on the direct costs of interventions for type 2 diabetes mellitus, hypertension, and cardiovascular disease in South Africa.

**Methods:**

PubMed® and Web of Science was searched for literature published between 01 and 1995 and 27 October 2022. Additionally, reference and citations lists of retrieved articles and experts were consulted. We also tracked reference lists of previous, related systematic reviews. Eligible publications were cost analyses of clinical interventions targeted at adults age 15 + reporting primary estimates of in- and out-of-hospital costs from a provider perspective. Costs were extracted and converted to 2021 US dollars, and article methodological and reporting quality was appraised using the 2013 CHEERS checklist.

**Results:**

Of the 600, 1,172 and 1,466 identified publications for type 2 diabetes mellitus, hypertension, and cardiovascular disease, respectively, 10, 12, and 17 met full inclusion criteria. 60% of articles reported cardiovascular disease costs, 52% were of good reporting quality, and 10%, 50%, and 39% of type 2 diabetes mellitus, hypertension and cardiovascular disease papers reported private-sector costs only. Hypertension drug costs ranged from $2 to $85 per person-month, while type 2 diabetes mellitus drug costs ranged between $57 and $630 per person-year (ppy). Diabetes-related complication treatment costs ranged from $55 for retinopathy treatment to $25,193 ppy for haemodialysis, while cardiovascular disease treatment costs were between $160 and $37,491 ppy. Drugs and treatment of complications were major cost drivers for hypertension and type 2 diabetes mellitus, while hospitalisation drove cardiovascular disease costs.

**Conclusion:**

The intervention costs of type 2 diabetes mellitus, hypertension and cardiovascular disease care have received more attention recently, particularly diabetes-related complications and cardiovascular disease. However, 39% of identified cardiovascular disease treatment costs used a private sector perspective, leaving significant research gaps in the public sector and the cheaper to treat hypertension and type 2 diabetes mellitus. This review fills an information gap regarding the intervention costs of these diseases in South Africa.

**Supplementary Information:**

The online version contains supplementary material available at 10.1186/s12889-022-14730-4.

## Introduction

In the most recently available estimates, from 2016, non-communicable diseases (NCD) led to 71% of all deaths and 75% of premature deaths (defined as deaths at ages 30 to 70) globally, with cardiovascular disease (CVD) accounting for 43·7% of all NCD deaths [1]. 77% of NCD deaths are in low- and middle-income countries (LMIC) [1]. In South Africa, in 2016, NCDs accounted for 51% of all deaths, including deaths due to CVD (19%), cancers (10%), diabetes (7%), chronic respiratory diseases (4%), and other NCDs (11%) [2]. NCD prevalence is set to increase in South Africa due to population ageing and improved management of infectious diseases, including HIV [3–5].

Diabetes mellitus (DM) is a chronic NCD that develops when the pancreas does not produce enough insulin or the body cannot effectively use insulin [6]. The number of people with DM globally has increased from 108 million in 1980 to 422 million in 2014, with prevalence increasing more rapidly in LMIC [6]. The International Diabetes Federation estimates that 463 million people aged between 20 and 79 years were living with DM in 2019 worldwide and 3 in 4 (79%) of these live in LMIC [7].

Other than gestational diabetes, there are 2 types of DM, Type 1 and Type 2. Most people with diabetes have Type 2 diabetes mellitus (T2DM), formerly known as non-insulin-dependent or adult-onset diabetes, accounting for over 90% of all cases in sub-Saharan Africa [8]. In South Africa, official government statistics suggested that DM was the second-highest natural cause of death between 2015 and 2017, out-ranked only by tuberculosis, and accounted for 5·7% of deaths in 2017 [9]. In 2000, it was estimated that 5·5% of South Africans aged over 30 years were living with diabetes, which was estimated to have increased to 9% in 2009 [10, 11].

If it goes untreated, DM can result in a plethora of micro- and macrovascular sequelae, including a two-fold increased risk of heart attacks and stroke and an increased risk of hypertension (HT), which increases the risk of adverse cardiovascular outcomes [12, 13]. Microvascular disease can lead to retinopathy, nephropathy, and neuropathy, while macrovascular disease can lead to cardiovascular complications such as coronary heart disease (CHD) and myocardial infarction (MI), cerebrovascular complications such as stroke or transient ischaemic attacks (TIA), and peripheral artery disease.

T2DM and HT frequently co-exist and are associated with an elevated risk of life-threatening CVD. HT, i.e. high or raised blood pressure, is approximately twice as likely to be found in people with DM than their non-diabetic counterparts [14]. Hypertensive people may be up to 2·5 times as likely to develop diabetes than normotensive individuals [14].

Though the underlying HT and DM are relatively inexpensive to treat and manage, treating their complications is costly because it requires high-level expertise and specialised equipment. A case-control study carried out in 22 countries found that a history of HT and DM are significant risk factors for all stroke, with odds ratios of 2·64 (99% CI 2·26 − 3·08) and 1.36 (99% CI 1·10 − 1·68), respectively [15]. Another case-control study carried out in 52 countries found that the odds ratio for acute MI is 13·01 (99% CI 10·69–15·83) for people with hypertension, diabetes and current smoking compared to those without [16].

Despite the large and growing size of their disease burden, little is known about the cost of T2DM, HT and CVD in South Africa. A recent review of the cost of DM in Africa only reported hospitalisation costs for South Africa, and studies identified did not specify the DM type [17]. Also, previous LMIC-focussed systematic reviews that considered the costs and/or the cost-effectiveness of interventions for DM, HT and/or CVD either did not include South Africa [18], only looked at some of the interventions for these NCDs [19–22], considered studies that had a household perspective or only analysed cost-effectiveness evidence (not costs) [23–25]. To our knowledge, literature on the direct costs of all types of clinical interventions for T2DM, HT and CVD in South Africa has not been previously reviewed.

In the context of South Africa moving to universal health coverage and government considering adopting a capitation fee financing model for primary health care, and given the predicted increase in the prevalence of these and other NCDs, decision-makers must understand the size and drivers of their costs to allocate scarce resources efficiently. This review aims to fill the evidence gap by summarising the direct costs of interventions for the screening, diagnosis, and treatment of T2DM (including its related complications), HT, and CVD, and the prevention of CVD, in South Africa. In addition, this review identifies factors that account for the differences in the costs reported by different publications and determines cost drivers.

## Methods

### Search strategy and selection criteria

Despite being related, the results of the cost analyses of these 3 disease areas are generally not reported within the same studies. We therefore conducted three separate systematic reviews of published literature, following PRISMA guidelines. We used PubMed® and Web of Science to identify literature published between 01 and 1995 and 27 October 2022. For each systematic review, our searches included a combination of MeSH and manually set search terms that referenced: the disease, intervention type, evaluation type (“cost*”, “econom*”, “financ*”, “resource*”, and “expenditure*”) as well as country (“South Africa”) (see Additional file 1 for search terms). Reference lists and citations of included articles were manually reviewed, and experts consulted to identify additional articles. Additionally, we tracked references of previous, related systematic reviews to ensure that relevant articles were not missed. Cost analyses covering interventions targeted at adults age 15 + were included if they met the inclusion criteria described in Additional file 2.

After removing duplicates, titles and abstracts were screened to identify relevant articles. At the initial screening, articles were included if they had any intervention related to the 3 disease areas and if they included any cost data. Secondary screening of full texts was carried out on the papers included in the first screening, documenting reasons for exclusion. Both screenings were carried out independently by 2 reviewers. Disagreements between reviewers were resolved through discussion, and a senior colleague was involved where consensus could not be reached.

### Data extraction

Data extraction was performed using a standardised Microsoft Excel form. The data were extracted by a single reviewer and verified by a second. Intervention costs were extracted as the cost per patient, and where this was not reported, it was calculated where possible. For interventions with ongoing resource use per person-year (ppy), per person-month (ppm) were extracted or calculated, and per-event costs were extracted for once-off procedures.

### Assessment of quality of evidence

The methodological and reporting quality of included publications was assessed using the 2013 ISPOR Consolidated Health Economics Evaluation Reporting Standards (CHEERS) 24-item checklist, which consists of the minimum set of important items to include when reporting economic evaluations [26]. While an updated version of the CHEERS checklist was released recently, we decided to use the version that was relevant during the period that the reviewed papers were written. Some of the publications included in the review were not economic evaluations by definition, and as such, some of the items in the checklist were not applicable. A scoring system was added to the checklist to grade the quality of each checklist item for each publication as follows: 0 (not considered), 1 (partially considered), 2 (fully considered) and not applicable (where an item on the checklist was not relevant to the publication). For each publication, the scores for all checklist items were summed up and divided by the maximum attainable total for all items relevant to that publication. Publications were assessed with and without items 23 and 24 of the CHEERS checklist (source of funding and conflict of interest), which are not required by some journals. Publications were categorised as follows: low quality (score less than 50%), moderate quality (score between 50% and 74%), and good quality (score of 75% or higher). The two reviewers assessed the quality of the included publications independently, with disagreements being resolved by consensus or the involvement of a senior colleague.

### Data analysis and synthesis

For comparability of costs across all publications, costs were converted into 2021 US dollars after updating them to 2021 South African Rand (ZAR) using International Monetary Fund inflation rates for South Africa [27, 28]. Similar interventions were grouped, and costs within these groupings analysed using a basic narrative approach to compare findings from different publications and possible reasons for cost variability.

## Results

**Fig. 1:**
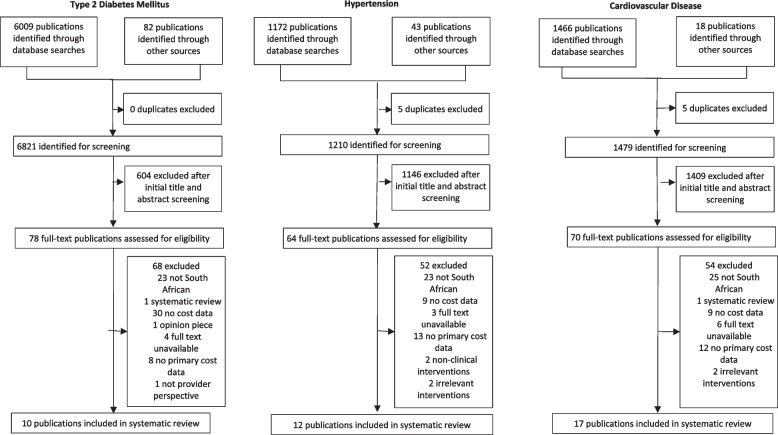
Study selection

Our initial PubMed® and Web of Science search identified 600, 1,172 and 1,466 publications for T2DM, HT and CVD, respectively and a further 82, 43, and 18 papers after the manual review (Fig. 1). Of 682, 1,210, and 1,479 articles, 68, 52, and 54 were excluded after full-text assessment, primarily because the publications were non-South African or had no cost or primary cost data.

Ten, twelve, and seventeen publications, a total of 29 publications, published between 1996 and 2019, met our inclusion criteria [29–57]. Of the included papers, two were Master’s theses [37, 38], 11 reported private-sector costs only [29, 31–33, 38, 44, 49, 50, 55–57], and 57% used an ingredients-based costing approach [29–32, 34–36, 38–41, 46–48, 50, 52, 54]. Study design varied across the publications, with just over a third modelling studies [32, 34–36, 38, 45, 48, 50, 53–55], 6 retrospective analyses [29, 33, 37, 40, 44, 51], 4 pharmacoeconomic analyses [31, 44, 49, 57], and 2 cost of illness studies [39, 52] (Table 1). Detailed results, including cost results by paper, can be found in Additional file 3, while Additional file 4 shows the CHEERS checklist per item for all included publications.

**Table 1:** Summary of publications included

Author, year of publication	Intervention(s)/Study focus	Study design	Cost data collection method	Provider	Reporting quality based on CHEERS checklist and % score^a^
**Hypertension -screening, monitoring, & management**					
Day [29] (1998)	Anti-hypertensive drugs	Retrospective survey	Ingredients-based	Private	Good
Edwards [30] (1998)	Anti-hypertensive drugs	Prospective study	Ingredients-based	Public	Moderate
Anderson [31](2000b)	Anti-hypertensive drugs	Pharmaco-economic analysis	Ingredients-based	Private	Good
Ker [32] (2008)	Anti-hypertensive and dyslipidaemia drugs	Single simulated-patient model	Ingredients-based	Private	Moderate
Makkink [33] (2014)	Anti-hypertensive drugs and CVD general costs	Retrospective cohort study	Top-down	Private	Moderate
Gaziano [34] (2014)	Monitoring and management of hypertension	Markov model	Ingredients-based	Public	Moderate
Gaziano [35] (2015a)	Anti-hypertensive drugs and screening costs	Microsimulation model	Ingredients-based	Public	Moderate
Basu [36] (2019)	Hypertension, T2DM, hyperlipidaemia, MI, stroke, heart failure, renal disease, diabetic retinopathy and neuropathy	Microsimulation model	Ingredients-based	Public	Good
**Type 2 diabetes mellitus - monitoring & management**					
Basu [36] (2019)	Hypertension, T2DM, hyperlipidaemia, MI, stroke, heart failure, renal disease, diabetic retinopathy and neuropathy	Microsimulation model	Ingredients-based	Public	Good
Nomame [37] (2012)	Management of T2DM and related complications	Retrospective cohort analysis	Bottom-up	Public	Good
Volmink [38] (2014)	Monitoring and management of T2DM	Probabilistic modelling	Ingredients-based	Private	Good
Erzse [39] (2019)	T2DM, retinopathy, cataracts, amputation, stroke, heart disease, and renal disease	Cost of illness study	Ingredients-based	Public	Moderate
**Screening for and treatment of diabetes-related complications**					
Erzse [39] (2019)	T2DM, retinopathy, cataracts, amputation, stroke, heart disease, and renal disease	Cost of illness study	Ingredients-based	Public	Moderate
Nomame [37] (2012)	Management of T2DM and related complications	Retrospective cohort analysis	Bottom-up	Public	Good
Ncube-Zulu [40] (2014)	Cerebrovascular disease, ophthalmic disease, CVD, renal disease, neurological disease, and peripheral vascular disease	Cross-sectional retrospective audit	Ingredients-based	Public	Moderate
Joannou [41] (1996)	Screening for diabetic retinopathy	N/S	Ingredients-based	N/S	Low
Khan [42] (2013)	Treatment of diabetic retinopathy and cataracts	Economic evaluation	Bottom-up	Public	Good
Pepper [43] (2007)	Hyperglycaemic emergency admissions	Prospective survey	Bottom-up	Public	Moderate
**CVD screening, prevention and treatment**					
Ker [32] (2008)	Anti-hypertensive and dyslipidaemia drugs	Single simulated-patient model	Ingredients-based	Private	Moderate
Makkink [33] (2014)	Anti-hypertensive drugs and CVD general costs	Retrospective cohort study	Top-down	Private	Moderate
Basu [36] (2019)	Hypertension, T2DM, hyperlipidaemia, MI, stroke, heart failure, renal disease, diabetic retinopathy and neuropathy	Microsimulation model	Ingredients-based	Public	Good
Ncube-Zulu [40] (2014)	Cerebrovascular disease, ophthalmic disease, CVD, renal disease, neurological disease, and peripheral vascular disease	Cross-sectional retrospective audit	Ingredients-based	Public	Moderate
Wessels [44] (2010)	Prevention and treatment of CVD	Retrospective pharmaco-economic analysis	Top-down	Private	Moderate
Gaziano [45] (2015b)	Treatment of dyslipidaemia	Microsimulation model	Bottom-up	Public	Good
Golovaty [46] (2018)	Screening for NCDs	Cross-sectional costing analysis	Ingredients-based	Public	Good
Laas [47] (2018)	Drugs for the prevention and treatment of CVD	Cross-sectional, observational study	Ingredients-based	Public	Moderate
Lin [48] (2019)	Prevention and treatment of CVD	Microsimulation model	Ingredients-based	Public	Good
Wessels [49] (2007)	MI, angina pectoris, stroke and TIA	Pharmacoeconomic assessment	N/S	Private	Moderate
Bergh [50] (2013)	Stroke, systemic embolism, and TIA	Markov modelling	Ingredients-based	Private	Good
Viljoen [51] (2014)	Stroke	Retrospective folder review	Bottom-up	Public	Good
Maredza [52] (2016)	Stroke	Cost of illness study	Ingredients-based	Public	Good
Manyema [53] (2016)	Stroke	Markov model	Top-down	Private & public	Good
Louw [54] (2019)	Stroke	Economic modelling	Ingredients-based	Public	Moderate
Anderson [55] (2000a)	Heart disease	Pharmaco-economic model	Top-down	Private	Moderate
Mabin [56] (2014)	Heart disease	Cost-comparison analysis	Top-down	Private	Good
Biccard [57] (2006)	Prevention and treatment of CVD	Pharmaco-economic analysis	Top-down	Private	Good

*Abbreviations:* *CVD* Cardiovascular Disease, *NCD* Non-communicable Disease, *MI* Myocardial Infarction, *TIA* Transient Ischaemic Attack

^a^Excluding items 23 & 24 of the CHEERS checklist

**Fig. 2:**
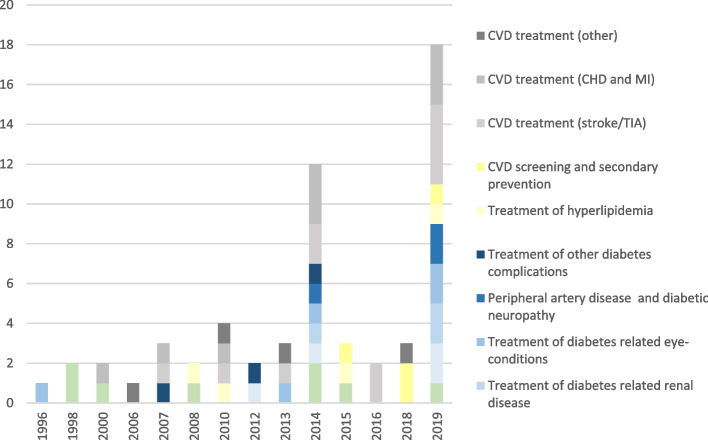
Number of papers by year of publication and intervention category

The included publications comprised 11 intervention groupings (Fig. 2). The number and reporting quality of publications increased over time, with 52% of papers rated as having good reporting quality (Table 1) [29, 31, 36–38, 42, 45, 46, 48, 50–53, 56, 57].

The number of papers published over time for T2DM and its related complications spiked in 2014 and 2019 [36–43]. The most documented T2DM complications were ophthalmic and renal disease, reported by 5 and 3 papers, respectively [36, 39–42]. 17 papers reported the costs of treating CVD, 41% reporting private sector costs only [33, 34, 36, 39, 40, 44, 47–57]. The number of CVD papers increased significantly from the 1990s to the 2010s, with the most costed CVDs being stroke and heart disease, reported by 11 and 9 publications, respectively [33, 34, 36, 39, 44, 48–56].

Eight papers reported the costs of monitoring and managing HT, with 50% of them using a private-sector provider perspective [29–36]. 75% of the identified papers reported the costs of antihypertensive drugs only, suggesting that they may be the main cost driver for managing HT [29–33, 35]. Private sector drug costs were in the range of $8 - $85 ppm [29, 31–33].Angiotensin-converting enzyme (ACE) inhibitors were 40% cheaper than angiotensin receptor blockers (ARBs) which were just under a quarter of the cost of angiotensin II receptor antagonists in the private sector [31, 33]. Fixed-dose combinations of an ACE inhibitor and a thiazide-like diuretic were more expensive than an ACE inhibitor alone, with the cost increasing with the addition of lipid-lowering drugs or a calcium channel blocker [32, 33]. Only one publication considered the lifetime costs of treating HT, estimated at approximately $1,860 [34].

For hypertensive drugs prescribed in the public sector, a 1998 publication reported the average cost of the most frequently prescribed drugs (diuretics, centrally acting agents and beta-blockers) at the last visit per month to be $2·34 per person (down from $3·09 at the first visit) [29]. A 2015 publication estimated the average cost of 3 commonly prescribed-hypertensive drugs (a channel blocker, a diuretic, and an ACE inhibitor) to be $7·96 ppy, almost a third of the cost reported by the 1998 paper [35]. The differences between these 2 public sector publications were in the type and distribution of hypertensive drugs and the sources of the costs. The cost of public-sector ACE inhibitors was 36% cheaper than the cost identified for the private sector.

Recently, there has been an increase in papers reporting costs of treatment of T2DM and its complications. For the treatment of T2DM, only one paper reported private-sector cost estimates at $630 and $523 ppy under a capitation model and usual practice, respectively [38]. In the public sector, annual T2DM drug costs ranged between $57 and $186 ppy [36]. The average cost of ACE inhibitors for hypertensive diabetic patients was slightly higher than for people with HT only [36]. Publications reported general costs of T2DM management of $77, $116, and $455 ppy, including some or all of the following items: labs, staff, consumables, equipment, and hospitalisation [6, 37, 39]. One of the publications found that, in 2018, oral antidiabetic drugs and insulin were major cost drivers accounting for 33% of total annual direct costs of managing diagnosed T2DM [39].

Six publications reported the costs of diabetes-related complications, including hyperglycaemic emergencies, amputation, and eye, kidney, neurological, and peripheral vascular disease, all using a public sector perspective [36, 37, 39, 40, 42, 43].

Treatment of diabetes-related renal disease was reported by 3 papers published in 2014 and 2019 [36, 39, 40]. One paper estimated the general costs of diabetes-related renal disease to be $3,585 ppy, included drugs, labs, and hospitalisation costs, and was almost double the estimated cost of renal disease for non-diabetic patients [40]. Another paper reported a ppy cost of $14,635 for 3 sessions of haemodialysis per week [36] while a third publication reported a ppy cost of $25,193 [39] for 2 sessions per week [39]. The inclusion of the cost of dialysis in 2 out of 3 of these papers may suggest that dialysis is a major cost driver for renal disease.

The cost of diabetes-related eye conditions was reported by 4 publications, of which 3 took a public sector perspective, and one did not state a perspective [36, 40–42]. Costs for screening for diabetic retinopathy were reported by 2 publications ($6·07 and $20 per person screened) with differences in screening mechanism and cost components included [41, 42]. The cost of treatment of diabetic retinopathy ppy was estimated at $55 by one paper [36], while another reported a per person cost of $132 for laser treatment of retinopathy and $169 for cataracts [42]. Another publication compared the ppy cost of treating ophthalmic disease in people with diabetes ($3,150) to treating ophthalmic disease in people without ($1,146) [40].

We found 3 papers reporting peripheral artery disease and diabetic neuropathy costs, with 2 estimating amputation costs, suggesting that amputation may be a cost driver for this category of diabetes-related complications [36, 39, 40]. One paper estimated a cost of amputation of $1,936 (including below-the-knee prosthesis), while another estimated the costs of minor and major amputation to be $1,656 and $3,379, respectively [36, 39]. The third publication found that the cost of treating peripheral vascular disease in patients with diabetes was 13% higher than those without [40].

We identified 3 papers for other diabetes complications: $866 per admission for hyperglycaemic emergency admissions; $224 ppy for drugs, labs, doctor consultations and emergency room visits; and costs ranging from $2,533 to $5,125 for treatment of cerebrovascular disease, neurological disease or CVD for patients with diabetes compared to costs for patients without diabetes which were between 10% and 37% cheaper [37, 40, 43].

Four papers reported the cost of treating hyperlipidaemia, and monthly costs for lipid-lowering drugs ranged from $2·58 - $33 in the public sector and $17 - $44 in the private sector [32, 36, 44, 45]. One paper reported public sector costs of labs and physician visits associated with hyperlipidaemia at $24 ppy [36].

The costs of CVD screening and secondary prevention were reported in 4 papers, all using a public sector perspective [35, 46–48]. One article compared paper- ($1·97 per person screened) to mobile phone-based ($1.01 per person screened) screening, while another considered integrating NCD to existing HIV screening, which cost an additional $4 per person screened [35, 46]. Drug costs for secondary prevention of CVD ranged between $24 and $54, with a fixed-dose combination pill estimated to cost $161 ppm [47, 48].

Eleven papers reported the cost of stroke and/or TIA, with 4 published in 2019 alone [34, 36, 39, 44, 48–54]. The costs of stroke treatment ranged between $1,359 - $3,521 ppy or event in the public sector, with hospitalisation costs ranging from $1,288 to $2,008 ppy. Private sector costs for the first stroke event were at least double the upper limit of the public sector cost range [44, 49]. One publication found that the biggest cost driver for acute stroke care was length of hospital stay [51]. This contribution of hospitalisation costs to the treatment of stroke was validated by two other publications which separated hospital costs from diagnostics and outpatient care costs [48, 52].

For treatment of (CHD) and MI, we identified 9 papers, 6 of them also reporting costs of stroke and/or TIA [33, 34, 36, 39, 44, 48–56]. Five of the publications reported only private sector costs [33, 44, 49, 55, 56]. Public sector drug costs ranged from $0.91 for aspirin to $114 for ACE inhibitors [36]. Also, in the public sector, treatment for non-MI heart disease ranged from $427 to $2,329 ppy and included drugs and hospitalisation, while the cost of MI was reported by 3 papers: $967 per event, $1,077 and $427 ppy [34, 36, 39, 48]. Private sector costs ranged from $1,292 per event for the hospitalisation costs of CHD, to $23,792 per event for angina pectoris associated costs and up to $33,662 per event for MI [44, 49, 55]. Costs generally increased with an increase in cost components, and notable increases were observed when hospitalisation costs were included [34, 36, 44, 48, 55, 56]. For publications that reported hospitalisation costs separately for MI and CHD, these ranged from $993 to $2,121 per event or ppy [36, 55].

Four papers reported on the costs of treatment of other CVDs, most of which were in the private sector [44, 47, 50, 57]. Public sector mean costs for inpatient and emergency care for patients on warfarin were estimated to be between $1,410 ppm and $160 for outpatient care [47]. The authors of this publication found that patients with non-valvular atrial fibrillation accounted for the largest proportion of these costs because of their patient admission costs and additional blood tests. In the private sector, treatment of CVD complications and adverse events were between $1,250 and $2,781 ppy, with costs varying according to their drug therapy before getting the complication, with patients on warfarin attracting the highest costs [50, 57]. Costs increased significantly when hospitalisation costs were included [44, 47, 50]. One publication compared outpatient to inpatient and emergency care and estimated mean ppm costs of $160 and $1,140, respectively [47].

## Discussion

To facilitate budget planning in a country moving towards universal health coverage, we conducted three systematic reviews to summarise available evidence on the direct costs of interventions for T2DM, HT, and CVD in South Africa while accounting for differences in results and determining cost drivers. Despite evidence of the high and increasing burden of these diseases [3–5], our review found limited analyses of the direct costs of disease interventions, and only 52% of publications were found to be of good reporting quality.

Across reviewed papers, we found that drugs were a major cost driver for managing HT and T2DM and hospitalisation for treating CVD. Only one publication considered the cost of treating people with both HT and T2DM and estimated that the average costs of ACE inhibitors for hypertensive diabetic patients was slightly higher than for people with HT only [36]. Most papers (60%) reported on the most expensive of the three included disease areas, CVD, with a significant increase in published articles over time. 10%, 50%, and 39% T2DM, HT, and CVD papers reported private-sector costs only.

Diabetes-related complications were more expensive to treat than managing uncomplicated diabetes. The complications with the most cost analyses were ophthalmic and renal disease, with T2DM almost doubling the cost of treating these diseases compared to treating non-diabetic counterparts. Amputation was the main cost driver of treatment for peripheral artery disease and diabetic neuropathy, while haemodialysis was a major cost driver for renal disease.

Cost differences across publications were due to variations in study designs, including the cost components considered and analytical methods used. There was a correlation between the number of cost components and the size of estimated costs. Results were most informative when they were disaggregated by cost categories, allowing for comparison. Unsurprisingly, private sector costs were much higher than public sector costs across interventions and analyses.

Our findings generally agree with results from previous reviews [17–22]. Similar to our findings, Mutyambizi et al. found that drug costs accounted for a significant proportion of direct costs for diabetes, even though their review did not differentiate between Type 1- and Type 2- DM and included all African countries [17]. Also similar to our findings, another review found that a large proportion of DM expenditure in LMICs was attributable to diabetes-related complications [18].

There are several limitations to our analysis. First, we only included literature published after 1995. However, given the distribution of publications identified, it is unlikely that we would have found many relevant publications from before 1995. Second, we only searched two databases and did not include grey literature. However, we also carried out manual citation and reference tracking, consulted experts, and included Master’s theses, making it less likely that we missed important publications. Third, results from the CHEERS checklist might not be replicable due to reviewer subjectivity. Fourth, we only considered studies from South Africa, which may not represent the majority of sub-Saharan Africa. However, this study offers a glimpse into the availability and nature of such data in the region. Fifth, the evidence base is somewhat sparse, and costs within intervention categories differed greatly in terms of cost components and costing methods making it difficult to summarise the evidence.

## Conclusion

In conclusion, while the intervention costs of T2DM, HT and CVD care have been historically neglected, more recently, more attention has been given to them- particularly their most expensive aspects, namely diabetes-related complications and CVD. Even so, 39% of publications reporting CVD treatment costs were from a private sector perspective, leaving significant research gaps in the public sector, which serves 80% of the South African population. Also, research focus needs to move to HT and T2DM, which, if well managed, can prevent deterioration to CVD and are cheaper to treat. Since drugs are a major cost driver for managing T2DM and DM, the future of NCD care depends on access to cheaper drugs, which may involve increased use of generic medication. This review, therefore, fills an information gap regarding the intervention costs of these diseases in South Africa.

## Supplementary Information

**Additional file 1.** PubMed® search strategies. 

**Additional file 2.** PICO criteria for inclusion.

**Additional file 3.** Results of the literature review.

**Additional file 4.** CHEERS checklist per item for all included studies.

## Data Availability

All data generated or analysed during this study are included in this published article [and its supplementary information files].

## Abbreviations

NCD: Non-communicable disease
CVD: Cardiovascular disease
LMIC: Low- and middle-income countries
DM: Diabetes mellitus
T2DM: Type 2 diabetes mellitus
HT: Hypertension
CHD: Coronary heart disease
MI: Myocardial infarction
TIA: Transient ischaemic attacks
Ppy: Per person-year
Ppm: Per person-month
PRISMA: Preferred Reporting Items for Systematic Reviews and Meta-Analyses
CHEERS: Consolidated Health Economics Evaluation Reporting Standards
