# Supplementary material for: The costs of interventions for type 2 diabetes mellitus, hypertension and cardiovascular disease in South Africa – a systematic literature review
Source: BMC Public Health. 2022 Dec 12;22:2321. doi: 10.1186/s12889-022-14730-4 (PMC9743545; doi:10.1186/s12889-022-14730-4)
Supplement: Supplementary file 1 — Additional file 1. PubMed® search strategies. [file 12889_2022_14730_MOESM1_ESM.docx]

**Appendix S1: PubMed^®^ search strategies**

***Type 2 diabetes mellitus search strategy***

("diabetes mellitus"[MeSH Major Topic] OR "diabetes mellitus, type 2"[MeSH Major Topic] OR "glucose intolerance"[MeSH Major Topic] OR "Hyperglycemia"[MeSH Major Topic] OR "Hypoglycemia"[MeSH Major Topic] OR "diabetes mellitus, type 2"[MeSH Major Topic] OR "blood glucose"[MeSH Major Topic] OR "glycated hemoglobin a"[MeSH Major Topic] OR "glycemic index"[MeSH Major Topic] OR "glycemic load"[MeSH Major Topic] OR "diabetes complications"[MeSH Major Topic] OR "prediabetic state"[MeSH Major Topic] OR "glucose intolerance"[MeSH Major Topic] OR "diabetic nephropathies"[MeSH Major Topic] OR "diabetic retinopathy"[MeSH Major Topic] OR "diabetic neuropathies"[MeSH Major Topic] OR ("diabetes mellitus, type 2"[MeSH Terms] OR "diabetes mellitus, type 2"[MeSH Terms] OR "insulin resistance"[MeSH Terms] OR "diabetes mellitus, type 2"[MeSH Terms] OR "glucose intolerance"[MeSH Terms]) OR ("diabetes mellitus"[Text Word] OR "diabetes mellitus type 2"[Text Word] OR "diabetes type 2"[Text Word] OR "T2DM"[Text Word] OR "diabetes type ii"[Text Word] OR "diabetes"[Text Word] OR "Hyperglycemia"[Text Word] OR "Hypoglycemia"[Text Word] OR (("prediabetic state"[MeSH Terms] OR ("prediabetic"[All Fields] AND "state"[All Fields]) OR "prediabetic state"[All Fields] OR ("pre"[All Fields] AND "diabetes"[All Fields]) OR "pre diabetes"[All Fields]) AND "prediabetes"[Text Word]) OR "prediabetic state"[Text Word] OR "pre diabetic state"[Text Word] OR "impaired glucose tolerance"[Text Word] OR "impaired fasting glucose"[Text Word] OR "potential diabetes"[Text Word] OR "pre diabetic stage"[Text Word] OR "latent diabetes"[Text Word] OR "prediabetic stage"[Text Word])) AND ("mass screening"[MeSH Major Topic] OR "biphasic insulins"[MeSH Major Topic] OR "insulin"[MeSH Major Topic] OR "glycemic control"[MeSH Major Topic] OR "metformin"[MeSH Major Topic] OR "glucose intolerance/blood"[MeSH Major Topic] OR "glucose intolerance/diagnosis"[MeSH Major Topic] OR "hyperglycemia/diagnosis"[MeSH Major Topic] OR "glycated hemoglobin a/analysis"[MeSH Major Topic] OR "thiazolidinediones"[MeSH Major Topic] OR "Glipizide"[MeSH Major Topic] OR "Glyburide"[MeSH Major Topic] OR "dipeptidyl peptidase iv inhibitors"[MeSH Major Topic] OR "glucagon like peptide 1"[MeSH Major Topic] OR ((("screen*"[Text Word] OR (("fingerprick"[All Fields] OR "fingerpricks"[All Fields]) AND "glucose test"[Text Word]) OR ((("finger s"[All Fields] OR "fingers"[MeSH Terms] OR "fingers"[All Fields] OR "finger"[All Fields]) AND ("prick"[All Fields] OR "pricked"[All Fields] OR "pricking"[All Fields] OR "pricks"[All Fields])) AND "glucose test"[Text Word]) OR "oral glucose tolerance test"[Text Word] OR "OGTT"[Text Word] OR "glucose tolerance test"[Text Word] OR "glycosylated hemoglobin"[Text Word] OR "HbA1c"[Text Word] OR "HbA1"[Text Word] OR "impaired fasting glucose"[Text Word] OR "IGT"[Text Word] OR "IFG"[Text Word] OR "fasting plasma glucose"[Text Word] OR "FPG"[Text Word] OR "oral glucose tolerance test"[Text Word] OR "OGTT"[Text Word] OR "random capillary blood glucose"[Text Word] OR "capillary blood glucose"[Text Word] OR "random blood glucose"[Text Word] OR "post prandial glucose"[Text Word] OR "impaired fasting glucose"[Text Word] OR "IFG"[Text Word] OR "impaired glucose tolerance"[Text Word] OR "IGT"[Text Word] OR "urine glucose test*"[Text Word] OR "treat*"[Text Word] OR "oral antidiabetic agent*"[Text Word] OR "oral antidiabetic drug*"[Text Word] OR "sulfonylurea*"[Text Word] OR "Glibenclamide"[Text Word] OR "Glimepiride"[Text Word] OR "metformin"[Text Word] OR "insulin"[Text Word] OR "glucagon like peptide 1"[Text Word] OR "biguanide*"[Text Word] OR "thiazolidinedione*"[Text Word] OR "Pioglitazone"[Text Word] OR "Rosiglitazone"[Text Word] OR "sulphonylurea*"[Text Word] OR "Glipizide"[Text Word] OR "Glyburide"[Text Word] OR "Insulin secretagogues"[All Fields]) AND "sitagliptin*"[Text Word]) OR "saxagliptin*"[Text Word] OR "Dpp-4"[Text Word] OR "Dpp-iv"[Text Word] OR "Liraglutide"[Text Word] OR "Exenatide"[Text Word] OR "complication*"[Text Word] OR "foot exam*"[Text Word] OR "eye exam*"[Text Word])) AND ("health care costs"[MeSH Major Topic] OR "health expenditures"[MeSH Major Topic] OR "health resources"[MeSH Major Topic] OR "health resources/economics"[MeSH Major Topic] OR "health resources/statistics and numerical data"[MeSH Major Topic] OR "costs and cost analysis"[MeSH Major Topic] OR "economics, medical"[MeSH Major Topic] OR "cost benefit analysis"[MeSH Major Topic] OR ("cost*"[Text Word] OR "econom*"[Text Word] OR "financ*"[Text Word] OR "resource*"[Text Word]) OR ("Health"[Text Word] AND "service"[Text Word] AND "cost"[Text Word]) OR ("resource"[Text Word] AND "use"[Text Word]) OR "expenditure*"[Title/Abstract]) AND ("south africa"[Text Word] OR "south africa*"[Text Word] OR "RSA"[Text Word] OR "southern africa"[Text Word] OR ("south africa"[MeSH Major Topic] OR "africa, southern"[MeSH Major Topic]) OR "Africa"[Text Word])

***Hypertension search strategy***

("hypertension"[MeSH Major Topic] OR ("blood pressure"[MeSH Major Topic] OR "blood pressure determination"[MeSH Major Topic] OR "arterial pressure"[MeSH Major Topic]) OR ("blood pressure"[Text Word] OR "Bloodpressure"[Text Word] OR "hypertens*"[Text Word] OR "prehypertens*"[Text Word] OR "arterial pressure"[Text Word] OR "diastolic pressure"[Text Word] OR "systolic pressure"[Text Word] OR "bp"[Text Word] OR "dbp"[Text Word] OR "hbp"[Text Word] OR "sbp"[Text Word])) AND ("screen*"[All Fields] OR "detect*"[All Fields] OR "monitor*"[All Fields] OR "angiotensin converting enzyme inhibit*"[Text Word] OR "beta antagonist*"[All Fields] OR "alpha adrenergic antagonist*"[Text Word] OR "alpha adrenergic receptor antagonist*"[Text Word] OR "adrenergic alpha antagonist*"[Text Word] OR "angiotensin receptor blocker*"[Text Word] OR "angiotensin ii receptor blocker*"[Text Word] OR "angiotensin receptor antagonist*"[Text Word] OR "angiotensin ii receptor antagonist*"[Text Word] OR "calcium channel blocker*"[Text Word] OR "calcium channel antagonist*"[Text Word] OR "angiotensin converting enzyme inhibitor*"[Text Word] OR "diuretic*"[All Fields] OR "fixed dose combination"[Text Word] OR "fixed dose combination"[Text Word] OR "treat*"[All Fields] OR "therap*"[All Fields] OR "complication*"[All Fields] OR "lower*"[All Fields] OR "fall*"[All Fields] OR "reduc*"[All Fields] OR "control*"[All Fields] OR ("Early"[Text Word] AND "diagnos*"[Text Word]) OR ("Early"[Text Word] AND "intervent*"[Text Word]) OR ("Angiotensin"[Text Word] AND ("receptor antagon*"[Text Word] OR "receptor block*"[Text Word])) OR ("Ace"[Text Word] AND "inhibit*"[Text Word]) OR ("Beta"[Text Word] AND ("adrenergic"[Text Word] OR "antagonist"[Text Word] OR "block*"[Text Word] OR "receptor"[Text Word])) OR (("Alpha"[Text Word] OR "alpha-adrenergic"[Text Word]) AND "block*"[Text Word]) OR ("sodium potassium chloride"[Text Word] AND ("cotransporter"[Text Word] OR "co-transporter"[Text Word] OR "symporter"[Text Word])) OR ("loop"[Text Word] AND "diuretic"[Text Word])) AND ("health care costs"[MeSH Major Topic] OR "health expenditures"[MeSH Major Topic] OR "health resources"[MeSH Major Topic] OR "health resources/economics"[MeSH Major Topic] OR "health resources/statistics and numerical data"[MeSH Major Topic] OR "costs and cost analysis"[MeSH Major Topic] OR "economics, medical"[MeSH Major Topic] OR "cost benefit analysis"[MeSH Major Topic] OR ("cost*"[Text Word] OR "econom*"[Text Word] OR "financ*"[Text Word] OR "budget*"[Text Word]) OR "service cost"[Text Word] OR "Resource use"[Text Word]) AND ("south africa"[MeSH Major Topic] OR "africa, southern"[MeSH Major Topic] OR ("Africa"[Text Word] OR "RSA"[Text Word] OR "SA"[Text Word]) OR ("Southern"[Text Word] AND "Africa"[Text Word]) OR ("South"[Text Word] AND "africa*"[Text Word]))

***Cardiovascular disease search strategy***

("cardiovascular diseases"[MeSH Major Topic] OR "stroke"[MeSH Major Topic] OR "coronary disease"[MeSH Major Topic] OR ("myocardial ischemia"[MeSH Major Topic] OR "coronary artery disease"[MeSH Major Topic]) OR "myocardial infarction"[MeSH Major Topic] OR "heart failure"[MeSH Major Topic] OR "heart arrest"[MeSH Major Topic] OR "stroke"[MeSH Major Topic] OR "carotid stenosis"[MeSH Major Topic] OR "arteriosclerosis"[MeSH Major Topic] OR "coronary thrombosis"[MeSH Major Topic] OR "angina, unstable"[MeSH Major Topic] OR "angina, stable"[MeSH Major Topic] OR ("CVD"[Text Word] OR "cardiovasc*"[Text Word] OR "coronar*"[Text Word] OR "heart*"[Text Word] OR "myocardi*"[Text Word] OR "cardiac*"[Text Word] OR "stroke*"[Text Word] OR "cerebrovasc*"[Text Word] OR "atherosclero*"[Text Word] OR "arteriosclero*"[Text Word] OR "cardiomyopath*"[Text Word] OR "angina*"[Text Word] OR "ischem*"[Text Word] OR "ischaem*"[Text Word] OR "Atrial fibrillation"[Text Word] OR "Auricular fibrillation"[Text Word] OR "Atrium fibrillation"[Text Word]) OR ("vascular*"[Text Word] AND "peripheral*"[Text Word]) OR ("vascular*"[Text Word] AND "disease*"[Text Word]) OR ("vascular*"[Text Word] AND "complication*"[Text Word])) AND ("drug therapy"[MeSH Major Topic] OR "diagnosis"[MeSH Major Topic] OR "diagnostic techniques and procedures"[MeSH Major Topic] OR "mass screening"[MeSH Major Topic] OR "early diagnosis"[MeSH Major Topic] OR "electrocardiography"[MeSH Major Topic] OR "electrocardiography, ambulatory"[MeSH Major Topic] OR "disease management"[MeSH Major Topic] OR "drug combinations"[MeSH Major Topic] OR "primary prevention"[MeSH Major Topic] OR "hydroxymethylglutaryl coa reductase inhibitors"[MeSH Major Topic] OR "hydroxymethylglutaryl coa reductases"[MeSH Major Topic] OR "anticholesteremic agents"[MeSH Major Topic] OR ("screen*"[Text Word] OR "detect*"[Text Word] OR "diagnos*"[Text Word] OR ("Early"[Text Word] AND "intervent*"[Text Word]) OR "prevent*"[Text Word] OR "treat*"[Text Word] OR "manage*"[Text Word] OR "statin*"[Text Word] OR "Aspirin"[Text Word] OR "Hypocholesterolemic agent"[Text Word] OR "electrocardiogram*"[Text Word] OR "electrocardiograph*"[Text Word] OR "ecg"[Text Word] OR "ekg"[Text Word] OR "holter"[Text Word] OR "event monitor*"[Text Word] OR "Treatment"[Text Word] OR "therap*"[Text Word]) OR ("hydroxymethylglutaryl*"[Text Word] AND "inhibitor*"[Text Word]) OR ("hmg coa*"[Text Word] AND "inhibit*"[Text Word])) AND ("health care costs"[MeSH Major Topic] OR "health expenditures"[MeSH Major Topic] OR "health resources"[MeSH Major Topic] OR "health resources/economics"[MeSH Major Topic] OR "health resources/statistics and numerical data"[MeSH Major Topic] OR "costs and cost analysis"[MeSH Major Topic] OR "economics, medical"[MeSH Major Topic] OR "cost benefit analysis"[MeSH Major Topic] OR ("cost*"[Text Word] OR "econom*"[Text Word] OR "service cost"[Text Word] OR "Resource use"[Text Word] OR "financ*"[Text Word] OR "budget*"[Text Word])) AND ("south africa"[MeSH Major Topic] OR "africa, southern"[MeSH Major Topic] OR ("Africa"[Text Word] OR "RSA"[Text Word] OR "SA"[Text Word]) OR ("South"[Text Word] AND "africa*"[Text Word]) OR ("Southern"[Text Word] AND "Africa"[Text Word]))
