# Supplementary material for: The costs of interventions for type 2 diabetes mellitus, hypertension and cardiovascular disease in South Africa – a systematic literature review
Source: BMC Public Health. 2022 Dec 12;22:2321. doi: 10.1186/s12889-022-14730-4 (PMC9743545; doi:10.1186/s12889-022-14730-4)
Supplement: Supplementary file 2 — Additional file 2. PICO criteria for inclusion. [file 12889_2022_14730_MOESM2_ESM.docx]

**Appendix S2: PICO criteria for inclusion**

| **Selection Criteria** | **Inclusion** | **Exclusion** |
| --- | --- | --- |
| Population | - Adults age 15+ (including those living with or at risk for T2DM, HT and/or CVD) | - Pregnant women - Children only |
| Intervention | - Any kind of clinical intervention for T2DM, HT, and/or CVD. Including their screening, diagnosis, and treatment. | - Non-clinical forms of treatment e.g. herbal treatments, non-traditional medicines, alternative treatments, or other methods not normally covered by the healthcare system |
| Comparators | - Not restricted (with or without a comparator) | - None |
| Outcomes | - Direct in-hospital and out-of-hospital costs. Direct costs were defined as actual costs due to resource use needed to deliver an intervention and included the cost of drugs, tests and hospital stays. - Costs estimated from actual patient level data, based on ingredients or expenditure - Costs estimated according to treatment pathways in clinical guidelines | - Only indirect costs reported such as productivity lost |
| Study type | - Any type of an economic analysis (including cost and cost-effectiveness or cost-utility analyses) reporting primary cost estimates based on a) patient level data, b) expenditure or c) ingredients, or a combination thereof, or calculating costs based on treatment pathways in clinical guidelines - Provider perspective (private or public). Provider costs were defined as the costs of delivering services as incurred by the health system, hospitals, clinics, programmes, medical aids, or other funders. If studies considered multiple perspectives, they were only included if they reported provider costs separately | - Systematic reviews to avoid double counting |
| Language | - Studies published in the English language | - Studies published in languages other than English |
| Other | - Studies available online - South Africa | - Studies that are not available online - From countries other than South Africa - Studies for which only an abstract is available |

Abbreviations: T2DM: Type 2 diabetes mellitus, HT: hypertension, CVD: cardiovascular disease
