# Supplementary material for: The costs of interventions for type 2 diabetes mellitus, hypertension and cardiovascular disease in South Africa – a systematic literature review
Source: BMC Public Health. 2022 Dec 12;22:2321. doi: 10.1186/s12889-022-14730-4 (PMC9743545; doi:10.1186/s12889-022-14730-4)
Supplement: Supplementary file 3 — Additional file 3. Results of the literature review. [file 12889_2022_14730_MOESM3_ESM.docx]

**Appendix S3: Results of the literature review**

| **No.** | **Author (year)** | **Objective** | **Target population, setting, level of care** | **Cost perspective** | **Costing method** | **Costing period*** | **Mean cost (2021 USD)** | **Range (2021 USD)** | **Cost items included** | | | | | | | | | | | |
| --- | --- | --- | --- | --- | --- | --- | --- | --- | --- | --- | --- | --- | --- | --- | --- | --- | --- | --- | --- | --- |
|  |  |  |  |  |  |  |  |  | All drugs | Disease drugs | Other drugs | Labs | Staff | Consumables | Overhead | Equipment | Capital | Transportation | Hospitalisation | Other |
| **Hypertension - monitoring, & management (private sector)** | | | | | | | | | | | | | | | | | | | | |
| 1 | Day^1^ (1998) | Examine the impact of managed care interventions for chronic illness prescriptions | N/S in all provinces; PHC | Provider (private) | Incremental financial cost, ingredients approach | 1995 | Treatment of hypertension (1st half of the year) Preferred provider cost: $33 Retail pharmacy cost: $64 Treatment of hypertension (2nd half of the year) Preferred provider cost: $32 Retail pharmacy cost: $64 per person month | N/A |  | x |  |  |  |  |  |  |  |  |  |  |
| 2 | Anderson^2^ (2000b) | Determine the cost-effectiveness of AT1 receptor blockers in the treatment of hypertension | N/A in N/S; PHC | Provider (private) | Incremental financial cost, ingredients approach | 2000 | Treatment of hypertension -angiotensin II receptor antagonists Candesartan: $49 Losartan: $49 Valsartan: $49 Irbesartan: $56 per person month | N/A |  | x |  |  |  |  |  |  |  |  |  |  |
| 3 | Ker^3^ (2008) | Investigate the effect and cost implications of drug treatment on the calculated absolute cardiovascular risk | Male smoker aged 56 years old; PHC | Provider (private) | Incremental financial cost, ingredients approach | 2006 | Treatment of hypertension Perindopril-indapamide (4.25mg/2.5mg): $23 Plus atorvastatin 10mg: $48 Plus amlopidine 5mg: $64 Plus bezafibrate: $85 per person month | N/A |  | x |  |  |  |  |  |  |  |  |  |  |
| 4 | Makkink^4^ (2014) | Assessing the costs of angiotensin-converting enzyme inhibitors (ACEI) versus angiotensin receptor blockers (ARB) | Registered members of the participating medical scheme in all provinces; PHC | Provider (private) | Incremental financial cost, top-down approach | 2010 - 2011 | Treatment of hypertension  Angiotensin-converting enzyme inhibitor group: $98 Angiotensin receptor blocker group: $134 Combined group: $264  per person-year | SE  $83 - $114  $108 - $160 $201 - $327 |  | x |  |  |  |  |  |  |  |  |  |  |
| **Hypertension - monitoring, & management (public sector)** | | | | | | | | | | | | | | | | | | | | |
| 5 | Edwards^5^ (1998) | Describe antihypertensive drug prescription pattern at a community health centre and costs of drug treatment and blood pressure control | Hypertensive patients with at least 2 medicine prescriptions during one year in urban Western cape; community health centre | Provider (public) | Incremental financial cost, ingredients approach | 1992 | Treatment of hypertension First visit: $3.09 Last visit: $2.34 per person month | N/A |  | x |  |  |  |  |  |  |  |  |  |  |
| 6 | Gaziano^6^ (2014) | Establish the cost effectiveness of training community health workers on hypertension as a medication adherence strategy | Adults aged 25-74 in all provinces; PHC | Provider (public) | Incremental financial cost, ingredients approach | (2013) | Treatment of hypertension Current standard: $1,854 CHW intervention: $1,860  per lifetime per person | N/A |  | x | x | x | x |  |  |  |  |  | x | Training, home visits & follow-up |
| 7 | Gaziano^7^ (2015a) | Evaluate the benefits, risks, and costs of two interventions for cardiovascular screening | Adults aged 35–74 with no prior history of stroke or ischemic heart disease; PHC | Provider (public) | Incremental financial cost, ingredients approach | (2014) | Treatment of hypertension: $7.96 per person-year | N/A |  | x |  |  |  |  |  |  |  |  |  |  |
| 8 | Basu^8^ (2019) | Estimate the economic implications of treatment scale-up under two alternative guidelines | Adults (>15), with data available on cardiovascular risk factors in all provinces; PHC & hospital | Provider (public) | Incremental financial cost, ingredients approach | 2012 | Monitoring and management of hypertension Physician visit, nurse visits & labs: $60 Thiazide: $47 ACE inhibitor: $63 Calcium channel blocker: $31 Beta-blocker: $0·91 per person-year | N/A |  | x |  | x | x |  |  |  |  |  |  |  |
| **Type 2 diabetes mellitus - monitoring & management (private sector)** | | | | | | | | | | | | | | | | | | | | |
| 9 | Volmink^9^ (2014) | Determine whether a private sector diabetes management programme (DMP) capitation model can be cost-effective in the public sector | N/S; PHC | Provider (private) | Incremental financial cost, ingredients approach | 2012 | Treatment and management of diabetes DMP capitation model: $630 per person-year | N/A |  | x |  | x | x | x | x |  |  |  |  | Training & incentive bonus |
|  |  |  |  |  |  |  | Treatment and management of diabetes Usual practice: $523 per person-year | N/A |  | x |  | x | x | x | x |  |  |  |  |  |
| **Type 2 diabetes mellitus - monitoring & management (public sector)** | | | | | | | | | | | | | | | | | | | | |
| 10 | Nomame^10^ (2012) | Determine the direct medical costs and predict the cost of diabetes mellitus and its complications at Groote Schuur hospital | Adults (>18) with diabetes mellitus; tertiary hospital | Provider (public) | Incremental financial cost, bottom-up approach | 2009 - 2010 | Management of diabetes  Mean: $193 Median: $186  per person over 20 months | SD  $22 - $316 N/A |  | x |  | x |  |  |  |  |  |  |  | Doctor consultations, dispensing fees, radiological & electrography procedures |
| 8 | Basu^8^ (2019) | Estimate the economic implications of treatment scale-up under two alternative guidelines | Adults (>15) with data available on cardiovascular risk factors; PHC & hospital | Provider (public) | Incremental financial cost, ingredients approach | 2012 | Treatment, monitoring and management of T2DM Physician & nurse visits & labs: $77 Metformin: $57 Sulfonylurea: $186 Insulin, basal: $105 Aspirin: $0.91 Statin: $31 ACE inhibitor: $35  per person-year | N/A |  | x |  | x | x |  |  |  |  |  |  |  |
| 11 | Erzse^11^ (2019) | Estimate the direct medical costs associated with type 2 diabetes mellitus | Adults (>15); PHC & hospital | Provider (public) | Incremental financial cost, ingredients approach | 2018 | Treatment, monitoring and management of T2DM: $455 per person-year | N/A |  | x |  | x | x | x |  | x |  |  | x |  |
| **Treatment of diabetes-related renal disease (public sector)** | | | | | | | | | | | | | | | | | | | | |
| 12 | Ncube-Zulu^12^ (2014) | Describe the difference in length and cost of hospitalisation between diabetic and non-diabetic patients with cerebrovascular, opthalmic, cardiovascular, renal, neurological and peripheral vascular diseases | Discharged patients with; cerebrovascular diseases, ophthalmic conditions, cardiovascular diseases, renal conditions, neurological diseases, or peripheral vascular diseases; tertiary hospital | Provider (public) | Incremental cost, ingredients approach | 2009 | Treatment of renal disease Patients with diabetes: $3,585 Patients without diabetes: $1,898  per person-year | SD $1,535 - $5,635 $191 - $3,605 | x |  |  | x |  |  |  |  |  |  | x |  |
| 8 | Basu^8^ (2019) | Estimate the economic implications of treatment scale-up under two alternative guidelines | Adults (>15) with data available on cardiovascular risk factors; PHC & hospital | Provider (public) | Incremental financial cost, ingredients approach | 2012 | Treatment of renal failure or end-stage renal disease A/V fistula surgery: $425 (once-off) Haemodialysis: $14,635 Physician visits & labs: $275 per person-year | *N/A* |  |  |  | x | x |  |  |  |  |  |  | AV fistula surgery & haemodialysis |
| 11 | Erzse^11^ (2019) | Estimate the direct medical costs associated with type 2 diabetes mellitus | Adults (>15); hospital | Provider (public) | Incremental financial cost, ingredients approach | 2018 | Renal imaging: $146 per person-year | N/A |  |  |  |  | x |  |  |  |  |  |  | Imaging |
|  |  |  |  |  |  |  | Renal biopsy: $33 per kidney | N/A |  |  |  |  | x |  |  |  |  |  |  | Biopsy |
|  |  |  |  |  |  |  | Haemodialysis: $25,193 per person-year | N/A |  |  |  |  |  |  |  |  |  |  |  | Haemo-  dialysis sessions |
| **Treatment of diabetes-related eye-conditions (unknown sector)** | | | | | | | | | | | | | | | | | | | | |
| 13 | Joannou^13^ (1996) | Compare; 60° mydriatic retinal photography, diabetes clinic doctors, formal ophthalmological assessment, and one or two 45° fields in the screening for diabetic retinopathy | Newly referred diabetes patients and those having an annual assessment for diabetic complications; PHC | N/S | Incremental financial cost, ingredients approach | (1995) | Screening for diabetic retinopathy using 60° mydriatic retinal photography: $6·07 per person screened | N/A |  |  |  |  | x |  |  |  |  |  |  | Price of film and processing |
| **Treatment of diabetes-related eye-conditions (public sector)** | | | | | | | | | | | | | | | | | | | | |
| 14 | Khan^14^ (2013) | Assess the cost-effectiveness of a diabetic retinopathy screening programme using a mobile fundal camera | Patients were members of a diabetic special care ‘‘club’’ in their respective community health centres; PHC | Provider (public) | Full financial cost, bottom-up approach | 2007 - 2010 | Screening for diabetic retinopathy using a mobile fundal camera: $20 Laser treatment in retinopathy: $132 Management of cataracts: $207  per person | NA |  |  | x |  | x | x | x | x | x | x |  | Transport costs to the patient, travel expenses for referral patient, & vehicle maintenance |
| 12 | Ncube-Zulu^12^ (2014) | Describe the difference in length and cost of hospitalisation between diabetic and non-diabetic patients with cerebrovascular, ophthalmic, cardiovascular, renal, neurological and peripheral vascular diseases | Discharged patients with; cerebrovascular diseases, ophthalmic conditions, cardiovascular diseases, renal conditions, neurological diseases, or peripheral vascular diseases; tertiary hospital | Provider (public) | Incremental cost, ingredients approach | 2009 | Treatment of ophthalmic disease Patients with diabetes: $3,150 Patients without diabetes: $1,146  per person-year | SD  $226 - $6,074 $160 - $2,133 | x |  |  | x |  |  |  |  |  |  | x |  |
| 8 | Basu^8^ (2019) | Estimate the economic implications of treatment scale-up under two alternative guidelines | Adults (>15) with data available on cardiovascular risk factors; PHC & hospital | Provider (public) | Incremental financial cost, ingredients approach | 2012 | Treatment of diabetic retinopathy: $55 per person-year | N/A |  |  |  |  | x |  |  |  |  |  |  | Ophthalmologist visit, ophthalmology care |
| **Peripheral artery disease and diabetic neuropathy (public sector)** | | | | | | | | | | | | | | | | | | | | |
| 12 | Ncube-Zulu^12^ (2014) | Describe the difference in length and cost of hospitalisation between diabetic and non-diabetic patients with cerebrovascular, opthalmic, cardiovascular, renal, neurological and peripheral vascular diseases | Discharged patients with; cerebrovascular diseases, ophthalmic conditions, cardiovascular diseases, renal conditions, neurological diseases, or peripheral vascular diseases; tertiary hospital | Provider (public) | Incremental cost, ingredients approach | 2009 | Treatment of peripheral vascular disease Patients with diabetes: $3,753 Patients without diabetes: $3,311  per person-year | SD  $1,429 - $6,077 $509 - $3,468 | x |  |  | x |  |  |  |  |  |  | x |  |
| 8 | Basu^8^ (2019) | Estimate the economic implications of treatment scale-up under two alternative guidelines | Adults (>15) with data available on cardiovascular risk factors; PHC & hospital | Provider (public) | Incremental financial cost, ingredients approach | 2012 | Treatment of diabetic neuropathy Physician & nurse visits & amitriptyline: $70 Acute ulcer: $532 ($478 once-off) Minor amputation: $1,709 ($1,656 once off) Major amputation: $3,853 ($3,379 once off) per person-year | *N/A* |  |  | x |  | x |  |  |  |  |  |  | Surgery theatre time, follow-up, physiotherapy for major amputation |
| 11 | Erzse^11^ (2019) | Estimate the direct medical costs associated with T2DM | Adults (>15); hospital | Provider (public) | Incremental financial cost, ingredients approach | 2018 | Amputation: $1,936 (once off) | N/A | x |  |  |  | x |  |  |  |  |  | x | Below knee prosthesis |
| **Treatment of other diabetes complications (public sector)** | | | | | | | | | | | | | | | | | | | | |
| 15 | Pepper^15^ (2007) | Determine reasons for and financial costs for hyperglycaemic emergency admissions to an urban, acute adult hospital | Patients admitted with hyperglycaemic emergency; hospital | Provider (public) | Incremental financial cost, bottom-up approach | 2005 | Treatment of hyperglycaemic emergency admissions Hyperglycaemic emergency admissions: $866 per admission | N/A | x |  |  | x | x |  | x |  | x |  |  | Imaging |
| 10 | Nomame^10^ (2012) | Determine the direct medical costs and predict the cost of diabetes mellitus and its complications at Groote Schuur hospital | Adults (>18) with diabetes mellitus; tertiary hospital | Provider (public) | Incremental financial cost, bottom-up approach | 2009 - 2010 | Management of diabetes mellitus complications Mean: $373 Median: $155  per person over 20 months | SD  $4 - $3,518 N/A | x |  |  | x |  |  |  |  |  |  | x | Doctor consultations, dispensing fees,  radiological & electrography  procedures, emergency room visits. |
| 12 | Ncube-Zulu^12^ (2014) | Describe the difference in length and cost of hospitalisation between diabetic and non-diabetic patients with cerebrovascular, ophthalmic, cardiovascular, renal, neurological and peripheral vascular diseases | Discharged patients with; cerebrovascular diseases, ophthalmic conditions, cardiovascular diseases, renal conditions, neurological diseases, or peripheral vascular diseases; tertiary hospital | Provider (public) | Incremental cost, ingredients approach | 2009 | Treatment of cerebrovascular disease Patients with diabetes: $4,996 Patients without diabetes: $3,164  Treatment of neurological disease Patients with diabetes: $5,125 Patients without diabetes: $4,603  per person-year | SD  $2,258 - $7,734 $1,091 - $5,238  $2,577 - $7,674 $834 - $8,373 | x |  |  | x |  |  |  |  |  |  | x |  |
| **Treatment of hyperlipidaemia (private sector)** | | | | | | | | | | | | | | | | | | | | |
| 3 | Ker^3^ (2008) | Investigate the effect and cost implications of drug treatment on the calculated absolute cardiovascular risk | Male smoker aged 56 years old; PHC | Provider (private) | Incremental financial cost, ingredients approach | 2006 | Treatment of hypertension & dyslipidaemia Lipid-lowering treatment 10 mg: $25 Lipid-lowering treatment 40 mg: $44 Hypertension and lipid-lowering treatment 10 mg: $48  per month | N/A |  | x |  | x |  |  |  |  |  |  |  |  |
| 16 | Wessels^16^ (2010) | Establish the healthcare cost impact of fenofibrate therapy for middle-aged T2DM patients at high risk of CVD | Patients aged 50 to 75 years with T2DM; PHC | Provider (private) | Full financial cost, top-down approach | 2008 | Lipid management Lipanthyl 200mg/day): $201 10mg simvastatin/day: $457  per person-year | N/A |  | x |  |  |  |  |  |  |  |  |  |  |
| **Treatment of hyperlipidaemia (public sector)** | | | | | | | | | | | | | | | | | | | | |
| 17 | Gaziano^17^ (2015b) | Investigate the health and economic impact of increasing prescription length from the standard monthly prescription | Adults (≥35) on a stable statin regimen for six months & no history of previous diagnosis of hypertension or diabetes or CVD; PHC | Provider (public) | Incremental financial cost, bottom-up approach | (2014) | Treatment of high cholesterol Simvastatin 20 mg (30-day prescription): $19 Simvastatin 20 mg (60-day prescription): $14 Simvastatin 20 mg (90-day prescription): $12 Atorvastatin 20 mg (30-day prescription): $33 Atorvastatin 20 mg (60-day prescription): $29  per person | N/A |  | x |  |  | x |  |  |  |  |  |  |  |
| 8 | Basu^8^ (2019) | Estimate the economic implications of treatment scale-up under two alternative guidelines | Adults (>15), with data available on cardiovascular risk factors in all provinces; PHC & hospital | Provider (public) | Incremental financial cost, ingredients approach | 2012 | Treatment of dyslipidaemia Physician visit & labs: $24 Statin: $31  per person-year | *N/A* |  |  | x | x | x |  |  |  |  |  |  |  |
| **CVD screening and secondary prevention (public sector)** | | | | | | | | | | | | | | | | | | | | |
| 7 | Gaziano^7^ (2015a) | Evaluate the benefits, risks, and costs of two interventions for cardiovascular screening | Adults (35–74) with no prior history of stroke or ischemic heart disease; community health workers | Provider (public) | Incremental financial cost, ingredients approach | (2014) | Screening of CVD Paper-based screening: $1·97 Mobile phone-based screening: $1·01  per person screened | N/A |  |  |  |  | x | x |  | x |  |  |  | Training |
| 18 | Golovaty^18^ (2018) | Determine the per-person incremental costs of integrating NCD screening and counselling to a home-based HIV counselling and testing program | Adults (≥18); HIV-/+; Previously enrolled in cohort study of HIV home-based testing and counselling (HTC) in 2012–2013 in rural KwaZulu Natal, South Africa; community-based | Provider (public) | Incremental financial cost, ingredients approach | Baseline costs - 2013  Incremental costs of HIV-NCD - 2016 | Screening (testing), counselling and referral to care  HIV rapid test & counselling: $11 HIV testing & NCD screening + counselling: $15  per person screened | N/A |  |  |  |  | x | x | x | x |  | x |  | Start-up, recurring meetings, and mobile phone data usage |
| 19 | Laas^19^ (2018) | Evaluate the cost-effectiveness and patients’ quality of care of warfarin therapy | Patients on warfarin; hospital | Provider (public) | Incremental financial cost, ingredients approaches | 2017 | Indication for warfarin - all Median: $24  Mean: $32  Indication for warfarin - non-valvular atrial fibrillation Median: $24  Mean: $35  Indication for warfarin - other Median: $24  Mean: $30  per person month | IQR: $23-27 SEM:$,2836  IQR: $2,328 SEM:$2,544   IQR: $2,327 SEM:$2,634 |  | x |  |  |  |  |  |  |  |  |  |  |
| 20 | Lin^20^ (2019) | Examine the cost-effectiveness and health effect of a polypill compared with current care for secondary prevention of atherosclerotic CVD | Adults (30–84) with established atherosclerotic CVD; PHC | Provider (public) | Incremental financial cost, ingredients approach | (2018) | Secondary prevention of atherosclerotic CVD Aspirin 75 mg: $49 Atenolol 50 mg: $32 Lisinopril 10 mg: $54 Simvastatin 40 mg: $28 Polypill: $161 per person month | N/A |  | x |  |  |  |  |  |  |  |  |  |  |
| **CVD treatment - stroke/transient ischaemic attack (private sector)** | | | | | | | | | | | | | | | | | | | | |
| 21 | Wessels^21^ (2007) | Assess the cost-effectiveness of eprosartan in secondary prevention of vascular disease | Hypertensive patients with previous cerebrovascular disease; hospital | Provider (private) | Incremental financial cost, unknown/not stated approach | (2006) | Treatment of stroke (first year): $11,002 Treatment of stroke (subsequent years): $4,555 Treatment of transient ischaemic attack (first year): $8,129 Treatment of transient ischaemic attack (subsequent years): $3,416  per event | N/A |  |  |  |  |  |  |  |  |  |  |  | NS |
| 16 | Wessels^16^ (2010) | Establish the healthcare cost impact of fenofibrate therapy for middle-aged type 2 diabetes patients at high risk of CVD | Patients aged 50 to 75 years with T2DM; hospital | Provider (private) | Full financial cost, top-down approach | 2008 | Treatment of stroke: $7,175 per event | N/A |  |  | x |  | x |  |  |  |  |  | x |  |
| 22 | Bergh^22^ (2013) | Estimate the cost-effectiveness and budgetary impact of using dabigatran for stroke prevention | Patients with atrial fibrillation; PHC | Provider (private) | Incremental financial cost, ingredients-based and top-down approaches | 2000 - 2007 | Prevention of stroke and systemic embolism Dabigatran: $6,746 Trial-like warfarin: $2,709  per year | N/A |  | x |  |  |  |  |  |  |  |  |  |  |
|  |  |  |  |  |  |  | Follow-up costs after a stroke Dabigatran: $26,427 Trial-like warfarin: $28,173  per year | N/A |  |  |  |  | x |  |  |  |  |  |  | Out -of-hospital |
| **CVD treatment - stroke/transient ischaemic attack (public sector)** | | | | | | | | | | | | | | | | | | | | |
| 12 | Ncube-Zulu^12^ (2014) | Describe the difference in length and cost of hospitalisation between diabetic and non-diabetic patients with cerebrovascular, ophthalmic, cardiovascular, renal, neurological and peripheral vascular diseases | Discharged patients with; cerebrovascular diseases, ophthalmic conditions, cardiovascular diseases, renal conditions, neurological diseases, or peripheral vascular diseases; tertiary hospital | Provider (public) | Incremental cost, ingredients approach | 2009 | Treatment of cardiovascular disease Patients with diabetes: $2,533 Patients without diabetes: $1,802 per person-year | SD  $785 - $4,281 $385 - $3,219 | x |  |  | x |  |  |  |  |  |  | x |  |
| 6 | Gaziano^6^ (2014) | Establish the cost-effectiveness of training community health workers on hypertension as a medication adherence strategy | Adults (25-74); PHC | Provider (public) | Incremental cost, ingredients approach | (2013) | Treatment of stroke: $1,359 per event | N/A |  | x |  | x | x |  |  |  |  |  | x |  |
| 23 | Viljoen^23^ (2014) | Determine the cost of acute stroke care and identify the cost drivers | All acute stroke patients admitted to general medical wards between January & 31 December 2012; hospital | Provider (public) | Incremental financial cost, bottom-up approach | 2012 | Treatment of Stroke Hospitalisation: $1,617 per person | N/A |  | x | x | x | x | x |  |  |  |  | x | Radiology |
| 24 | Maredza^24^ (2016) | Estimate the cost of stroke care in rural South Africa | Patients on a hospital-based stroke register; hospital | Provider (public) | Incremental financial cost, ingredients approach | 2012 | Diagnostic tests for stroke: $34 per event | N/A |  |  |  | x |  |  |  |  |  |  |  | Blood sugar, electrocardiogram, and CT Scan |
|  |  |  |  |  |  |  | Hospitalisation for stroke: $1,706 per event | N/A |  |  |  |  |  |  |  |  |  |  | x |  |
|  |  |  |  |  |  |  | Treatment and management of stroke: $204 per event | N/A |  | x | x | x |  |  |  |  |  |  |  |  |
| 8 | Basu^8^ (2019) | Estimate the economic implications of treatment scale-up under two alternative guidelines | Adults (>15), with data available on cardiovascular risk factors in all provinces; PHC & hospital | Provider (public) | Incremental financial cost, ingredients approach | 2012 | Treatment of stroke Hospitalisation & follow-up visits: $2,076 ($2,008 once off) Aspirin: $0·91 Statin: $31  per person-year | *N/A* |  |  | x | x | x |  |  |  |  | x | x |  |
| 11 | Erzse^11^ (2019) | Estimate the direct medical costs associated with T2DM | Adults (>15); hospital | Provider (public) | Incremental financial cost, ingredients approach | 2018 | Treatment of stroke: $2,673  per person-year | N/A |  |  |  |  | x |  |  |  |  |  |  | Facility fees |
| 25 | Louw^25^ (2019) | Estimate the current economic value of stroke rehabilitation | Previously employed stroke survivors in N/S; hospital | Provider (public) | Incremental financial cost, ingredients approach | (2018) | Stroke rehabilitation & re-assessment Rehabilitation: $321 Re-assessment: $46  per person | N/A |  |  |  |  | x |  |  |  |  |  |  | Other |
| 20 | Lin^20^ (2019) | Examine the cost-effectiveness and health effect of a polypill compared with current care for secondary prevention of atherosclerotic CVD | Adults (30–84) with established atherosclerotic CVD; PHC | Provider (public) | Incremental financial cost, ingredients approach | (2018) | Stroke - outpatient care: $64 Stroke - admission to hospital: $1,288  per person-year | N/A |  | x |  |  |  |  |  |  |  |  | x | Out-patient care excl drugs |
| **CVD treatment - stroke/transient ischaemic attack (private and public sector)** | | | | | | | | | | | | | | | | | | | | |
| 26 | Manyema^26^ (2016) | Estimate the impact of a sugar-sweetened beverage tax on the burden of stroke | Adults (>15 years); PHC & hospital | Provider (private & public) | Incremental financial cost, top-down approach | 2012 | Stroke-related costs Age 15-24: $3,052 Age 25-34: $1,775 Age 35-44: $2,582 Age 45-54: $3,307 Age 55-64: $3,521 Age 65-84+: $3,434  per person | N/A |  |  |  |  |  |  |  |  |  |  |  | N/S |
| **CVD treatment - coronary heart disease and myocardial infarction (private sector)** | | | | | | | | | | | | | | | | | | | | |
| 27 | Anderson^27^ (2000a) | Explain the economic impact of the use of ramipril in South Africa for post-myocardial infarction patients with heart failure | NS; hospital | Provider (private) | Incremental financial cost, top-down approach | (1999) | Management of heart disease Ramipril: $704 per person year | N/A |  | x |  |  |  |  |  |  |  |  |  |  |
|  |  |  |  |  |  |  | Treatment and monitoring of heart disease Hospital account: $1,292 per event | N/A |  |  |  |  |  |  |  |  |  |  | x |  |
|  |  |  |  |  |  |  | Monitoring of heart disease Professional fee: $456 per event | N/A |  |  |  |  | x |  |  |  |  |  |  | Professional fee |
| 21 | Wessels^21^ (2007) | Assess the cost-effectiveness of eprosartan in secondary prevention of vascular disease | Hypertensive patients with previous cerebrovascular disease; hospital | Provider (private) | Incremental financial cost, unknown/not stated approach | (2006) | Treatment of myocardial infarction (first event, first year): $33,622 Treatment of myocardial infarction (first event, subsequent years): $11,820 Treatment of myocardial infarction (subsequent events, first year): $15,780 Treatment of myocardial infarction (subsequent events, subsequent years): $1,431 Treatment of angina pectoris (first year): $23,792 Treatment of angina pectoris (subsequent years): $8,571  per event | N/A |  |  |  |  |  |  |  |  |  |  |  | NS |
| 16 | Wessels^16^ (2010) | Establish the healthcare cost impact of fenofibrate therapy for middle-aged type 2 diabetes patients at high risk of CVD | Patients aged 50 to 75 years with T2DM; hospital | Provider (private) | Full financial cost, top-down approach | 2008 | Treatment of non-fatal MI: $14,480 Coronary angiography: $2,267 Coronary revascularisation: $16,768  per event | N/A |  |  | x |  | x |  |  |  |  |  | x |  |
| 4 | Makkink^4^ (2014) | Assessing the costs of angiotensin-converting enzyme inhibitors (ACEI) versus angiotensin receptor blockers (ARB) | Registered members of the participating medical scheme; hospital | Provider (private) | Incremental financial cost, top-down approach | 2010-2011 | Treatment of cardiovascular disorders/complications Angiotensin-converting enzyme inhibitor group: $737 Angiotensin receptor blocker group: $979 Combined group: $2,726  per person-year | SE $721 - $752 $953 - $1,005 $2,664 - $2,789 |  | x | x |  |  |  |  |  |  |  |  | N/S |
| 28 | Mabin^28^ (2014) | Evaluate the costs and benefits of transcatheter aortic valve implantation (TAVI) and conventional aortic valve replacement (cAVR) | High risk aortic stenosis patients who could have had TAVI vs. those who underwent TAVI in Free State, Gauteng, Mpumalanga & Western Cape; hospital | Provider (private) | Incremental financial cost, top-down approach | 2009 - 2011 | Treatment of aortic stenosis Transcatheter aortic valve implantation (TAVI): $37,491 Conventional aortic valve replacement (cAVR): $23,902  per person | SD $32,138 - $42,843 $14,136 - $33,669 |  |  |  |  |  |  |  |  |  |  | x |  |
| **CVD treatment - coronary heart disease and myocardial infarction (public sector)** | | | | | | | | | | | | | | | | | | | | |
| 6 | Gaziano^6^ (2014) | Establish the cost effectiveness of training community health workers on hypertension as a medication adherence strategy | Adults (25-74); PHC | Provider (public) | Incremental cost, ingredients approach | (2013) | Treatment of myocardial infarction: $967 per event | N/A |  | x |  | x | x |  |  |  |  |  | x |  |
| 8 | Basu^8^ (2019) | Estimate the economic implications of treatment scale-up under two alternative guidelines | Adults (>15), with data available on cardiovascular risk factors in all provinces; PHC & hospital | Provider (public) | Incremental financial cost, ingredients approach | 2012 | Treatment of myocardial infarction Hospitalisation & follow-up visits: $1,077 ($993 once off) Aspirin: $0·91 Beta-blocker: $33 Statin: $31 ACE inhibitor: $114  per person-year | *N/A* |  |  | x | x | x |  |  |  |  | x | x |  |
|  |  |  |  |  |  |  | Treatment of congestive heart failure Hospitalisation & follow-up visits: $2,329 ($2,121 once off) Diuretic: $56 Beta-blocker: $30 Spironolactone: $8·21  per person-year | *N/A* |  |  | x | x | x |  |  |  |  | x | x | Echocardiogram |
| 11 | Erzse^11^ (2019) | Estimate the direct medical costs associated with type 2 diabetes mellitus | Adults (>15); hospital | Provider (public) | Incremental financial cost, ingredients approach | 2018 | Treatment ischaemic heart disease: $1,662 per person-year | N/A |  |  |  |  | x |  |  |  |  |  |  | Facility fees |
| 20 | Lin^20^ (2019) | Examine the cost-effectiveness and health effect of a polypill compared with current care for secondary prevention of atherosclerotic CVD | Adults (30–84), with established atherosclerotic CVD; PHC | Provider (public) | Incremental financial cost, ingredients approach | (2018) | Treatment of coronary heart disease: $42 per person-year | N/A |  | x |  |  |  |  |  |  |  |  |  | Out-patient care minus drugs |
|  |  |  |  |  |  |  | Treatment of CVD Treatment of myocardial infarction: $427 per person-year | N/A |  | x |  |  | x |  |  |  |  |  | x |  |
| **CVD treatment -other (private sector)** | | | | | | | | | | | | | | | | | | | | |
| 30 | Biccard^29^ (2006) | A cost-effectiveness analysis of the use of beta-blocker and statin therapy in high-risk non-cardiac surgery and vascular surgery patients | Adults (61-75) in all provinces; hospital | Provider (private) | Incremental financial cost, top-down approach | 2004 | Prevention & treatment of cardiovascular complications and adverse events Peri-operative beta-blocker therapy: $2,007 No beta-blocker therapy: $2,153 per person-year | N/A |  | x |  |  |  |  |  |  |  |  |  | CVD complications & adverse drug events |
|  |  |  |  |  |  |  | Prevention & treatment of cardiovascular complications and adverse events  Peri-operative statin therapy: $1,250 No statin therapy: $1,558 per person-year | N/A |  | x |  |  |  |  |  |  |  |  |  | CVD complications |
| 16 | Wessels^16^ (2010) | Establish the healthcare cost impact of fenofibrate therapy for middle-aged type 2 diabetes patients at high risk of CVD | Patients aged 50 to 75 years with T2DM; hospital | Provider (private) | Full financial cost, top-down approach | 2008 | Non-coronary revascularisation: $2,586 per event | N/A |  |  | x |  | x |  |  |  |  |  | x |  |
| 22 | Bergh^22^ (2013) | Estimate the cost-effectiveness and budgetary impact of using dabigatran for stroke prevention | Patients with atrial fibrillation; hospital | Provider (private) | Incremental financial cost, ingredients-based and top-down approaches | 2000 - 2007 | Treatment of stroke, systemic embolism, ischaemic attack or atrial fibrillation & adverse events Dabigatran: $2,618 Trial-like warfarin: $2,781 per person-year | N/A |  |  |  |  |  |  |  |  |  |  | x |  |
| **CVD treatment -other (public sector)** | | | | | | | | | | | | | | | | | | | | |
| 19 | Laas^19^ (2018) | Evaluate the cost-effectiveness and patients’ quality of care of warfarin therapy | Patients on warfarin; hospital | Provider (public) | Incremental financial cost, ingredients approaches | 2017 | Inpatient and emergency care Median: $978  Mean: $1,140  per person month | IQR: $434 - $1,846 SEM: $667 - $1,612 |  | x | x | x |  |  |  |  |  |  | x | Other investigations |
|  |  |  |  |  |  |  | Outpatient care  Median: $142  Mean: $160  per person month | IQR: $136 - $160 SEM: $156 - $163 |  | x |  | x |  |  |  |  |  |  |  | Outpatient and emergency room visits |

*Where the costing period was not reported, it was assumed to be the year before publication and bracketed.

Abbreviations: T2DM: Type 2 diabetes mellitus. CVD: Cardiovascular disease. SD: Standard deviation. SE: Standard error. SEM: Standard error of the mean. IQR: Interquartile range.

**References**

1. Day K, Booyens S. The cost-effectiveness of managed care regarding chronic medicine prescriptions in a selected medical scheme. Curationis. 1998;21(4):65–70.

2. Anderson AN, Wessels F, Moodley I, Kropman K. AT1 receptor blockers--cost-effectiveness within the South African context. S Afr Med J [Internet]. 2000 May;90(5):494–8. Available from: NL_INC

3. Ker JA, Oosthuizen H, Rheeder P. Decision-making using absolute cardiovascular risk reduction and incremental cost-effectiveness ratios: A case study. Cardiovasc J Afr. 2008;19(2):97–101.

4. Makkink JL, Greeff OBW. Angiotensin converting enzyme inhibitors v. angiotensin receptor blockers in the management of hypertension: a funder’s perspective. S Afr Med J [Internet]. 2014 Apr;104(4):292–4. Available from: NL_EXC

5. Edwards PR, Lunt DW, Fehrsen GS, Lombard CJ, Steyn K. Improving cost-effectiveness of hypertension management at a community health centre. S Afr Med J [Internet]. 1998 May;88(5):549–54. Available from: NL_INC

6. Gaziano TA, Bertram M, Tollman SM, Hofman KJ. Hypertension education and adherence in South Africa: a cost-effectiveness analysis of community health workers. BMC Public Health [Internet]. 2014 Mar;14:240. Available from: NL_INC

7. Gaziano T, Abrahams-Gessel S, Surka S, Sy S, Pandya A, Denman CA, et al. Cardiovascular disease screening by community health workers can be cost-effective in low- resource countries. Health Aff [Internet]. 2015;34(9):1538–45. Available from: https://www.scopus.com/inward/record.uri?eid=2-s2.0-84942309984&doi=10.1377%2Fhlthaff.2015.0349&partnerID=40&md5=cc210ab65a3a2ef65981ffa0e53131c1

8. Basu S, Wagner RG, Sewpaul R, Reddy P, Davies J. Implications of scaling up cardiovascular disease treatment in South Africa: a microsimulation and cost-effectiveness analysis. Lancet Glob Heal [Internet]. 2019 Feb;7(2):e270–80. Available from: NL_INC

9. Volmink HC, Bertram MY, Jina R, Wade AN, Hofman KJ. Applying a private sector capitation model to the management of type 2 diabetes in the South African public sector: A cost-effectiveness analysis. BMC Health Serv Res [Internet]. 2014;14(1). Available from: https://www.scopus.com/inward/record.uri?eid=2-s2.0-84908086237&doi=10.1186%2F1472-6963-14-444&partnerID=40&md5=b35aa3f3e5075226b7573520ec168376

10. Nomame S. Development of a Model To Predict the Cost of Management of Diabetes Mellitus and Its Complications At Groote Schuur Hospital.

11. Erzse A, Stacey N, Chola L, Tugendhaft A, Freeman M, Hofman K. The direct medical cost of type 2 diabetes mellitus in South Africa: a cost of illness study. Glob Health Action. 2019;12(1):1–9.

12. Ncube-Zulu T, Danckwerts MP. Comparative hospitalization cost and length of stay between patients with and without diabetes in a large tertiary hospital in Johannesburg, South Africa. Int J Diabetes Dev Ctries. 2014;34(3):156–62.

13. Joannou J, Kalk WJ, Mahomed I, Ntsepo S, Berzin M, Joffe BI, et al. Screening for diabetic retinopathy in South Africa with 60° retinal colour photography. J Intern Med. 1996;239(1):43–7.

14. Khan T, Bertram MY, Jina R, Mash B, Levitt N, Hofman K. Preventing diabetes blindness: Cost effectiveness of a screening programme using digital non-mydriatic fundus photography for diabetic retinopathy in a primary health care setting in South Africa. Diabetes Res Clin Pract [Internet]. 2013;101(2):170–6. Available from: https://www.scopus.com/inward/record.uri?eid=2-s2.0-84881312079&doi=10.1016%2Fj.diabres.2013.05.006&partnerID=40&md5=0091ff0f25f877f5b2b785c4a63da005

15. Pepper DJ, Levitt NS, Cleary S, Burch VC. Hyperglycaemic emergency admissions to a secondary-level hospital - An unnecessary financial burden. South African Med J. 2007;97(10 I):963–7.

16. Wessels F. Is fenofibrate a cost-saving treatment for middle-aged individuals with type 2 diabetes? A South African private-sector perspective. Cardiovasc J Afr. 2010;21(1):43–6.

17. Gaziano T, Cho S, Sy S, Pandya A, Levitt NS, Steyn K. Increasing prescription length could cut cardiovascular disease burden and produce savings in south africa. Health Aff. 2015;34(9):1578–85.

18. Golovaty I, Sharma M, Van Heerden A, Van Rooyen H, Baeten JM, Celum C, et al. Cost of integrating noncommunicable disease screening into home-based HIV testing and counseling in South Africa. J Acquir Immune Defic Syndr. 2018;78(5):522–6.

19. Laäs DJ, Naidoo M. An evaluation of warfarin use at an urban district-level hospital in Kwazulu-natal Province, South Africa. South African Med J [Internet]. 2018;108(12):1046–50. Available from: https://www.scopus.com/inward/record.uri?eid=2-s2.0-85050767922&doi=10.7196%2FSAMJ.2018.v108i12.13256&partnerID=40&md5=d14555f575a953b532f3f7d6e9c32910

20. Lin JK, Moran AE, Bibbins-Domingo K, Falase B, Pedroza Tobias A, Mandke CN, et al. Cost-effectiveness of a fixed-dose combination pill for secondary prevention of cardiovascular disease in China, India, Mexico, Nigeria, and South Africa: a modelling study. Lancet Glob Heal. 2019;7(10):e1346–58.

21. Wessels F. Eprosartan in secondary prevention of stroke: The economic evidence. Cardiovasc J South Africa. 2007;18(2):95–6.

22. Bergh M, Marais CA, Miller-Jansön H, Salie F, Stander MP. Economic appraisal of dabigatran as first-line therapy for stroke prevention in atrial fibrillation. S Afr Med J [Internet]. 2013 Feb;103(4):241–5. Available from: nl_inc

23. Viljoen CA, Dalmeyer L, de Villiers L. Cost of acute stroke care in South Africa. Stroke. 2013;8(1):3–4.

24. Maredza M, Chola L. Economic burden of stroke in a rural South African setting. eNeurologicalSci [Internet]. 2016;3:26–32. Available from: https://www.scopus.com/inward/record.uri?eid=2-s2.0-84976633598&doi=10.1016%2Fj.ensci.2016.01.001&partnerID=40&md5=de78610df005a58dfa42b8561ca6fb64

25. Louw Q, Twizeyemariya A, Grimmer K, Leibbrandt D. Estimating the costs and benefits of stroke rehabilitation in South Africa. J Eval Clin Pract [Internet]. 2020;26(4):1181–7. Available from: https://doi.org/10.1111/jep.13287

26. Manyema M, Veerman LJ, Tugendhaft A, Labadarios D, Hofman KJ. Modelling the potential impact of a sugar-sweetened beverage tax on stroke mortality, costs and health-adjusted life years in South Africa. BMC Public Health [Internet]. 2016 May;16:405. Available from: nl_inc

27. Nixon Anderson A, Moodley I, Kropman K. A South African pharmaco-economic analysis of the acute infarction ramipril efficacy (AIRE) Study. Cardiovasc J South Africa. 2000;11(2):89–94.

28. Mabin TA, Condolfi P. An analysis of real-world cost-effectiveness of TAVI in South Africa. Cardiovasc J Afr [Internet]. 2014;25(1):21–6. Available from: nl_inc

29. Biccard BM, Sear JW, Foëx P. The pharmaco-economics of peri-operative beta-blocker and statin therapy in South Africa. S Afr Med J [Internet]. 2006 Nov;96(11):1199–202. Available from: nl_inc
