# Supplementary material for: The costs of interventions for type 2 diabetes mellitus, hypertension and cardiovascular disease in South Africa – a systematic literature review
Source: BMC Public Health. 2022 Dec 12;22:2321. doi: 10.1186/s12889-022-14730-4 (PMC9743545; doi:10.1186/s12889-022-14730-4)
Supplement: Supplementary file 4 — Additional file 4. CHEERS checklist per item for all included studies. [file 12889_2022_14730_MOESM4_ESM.docx]

**Appendix S4: CHEERS checklist per item for all included studies**

| **Item No./Section/item** | Day^1^ (1998) | Edwards^2^ (1998) | Anderson^3^ (2000b) | Ker^4^ (2008) | Makkink^5^ (2014) | Gaziano^6^ (2014) | Gaziano^7^ (2015a) | Basu^8^ (2019) | Nomame^9^ (2012) | Volmink^10^ (2014) | Erzse^11^ (2019) | Ncube-Zulu^12^ (2014) | Joannou^13^ (1996) | Khan^14^ (2013) | Pepper^15^ (2007) | Wessels^16^ (2010) | Gaziano^17^ (2015b) | Golovaty^18^ (2018) | Laas^19^ (2018) | Lin^20^ (2019) | Wessels^21^ (2007) | Bergh^22^ (2013) | Viljoen^23^ (2014) | Maredza^24^ (2016) | Manyema^25^ (2016) | Louw^26^ (2019) | Anderson^27^ (2000a) | Mabin^28^ (2014) |  | Biccard^29^ (2006) | % of papers reporting item |
| --- | --- | --- | --- | --- | --- | --- | --- | --- | --- | --- | --- | --- | --- | --- | --- | --- | --- | --- | --- | --- | --- | --- | --- | --- | --- | --- | --- | --- | --- | --- | --- |
| **Title and abstract** | | | | | | | | | | | | | | | | | | | | | | | | | | | | | | | |
| 1.Title | Y | Y | Y | Y | P | Y | Y | Y | Y | Y | Y | P | N | Y | P | P | P | Y | P | Y | P | Y | Y | P | Y | Y | Y | Y |  | Y | 97% |
| 2.Abstract | P | P | Y | P | Y | P | P | P | Y | Y | P | P | P | P | P | Y | P | P | P | Y | Y | Y | Y | Y | P | P | P | Y |  | Y | 100% |
| **Introduction** | | | | | | | | | | | | | | | | | | | | | | | | | | | | | | | |
| 3.Background and objectives | Y | Y | Y | Y | Y | Y | Y | Y | Y | Y | Y | P | Y | Y | Y | Y | Y | Y | Y | Y | P | Y | Y | Y | Y | Y | Y | Y |  | Y | 100% |
| **Methods** | | | | | | | | | | | | | | | | | | | | | | | | | | | | | | | |
| 4.Target population and subgroups | Y | Y | Y | P | Y | P | Y | Y | Y | P | P | P | Y | P | Y | P | Y | Y | Y | Y | Y | P | Y | Y | Y | Y | Y | Y |  | Y | 100% |
| 5.Setting and location | Y | Y | P | P | Y | P | Y | Y | Y | Y | Y | P | N | Y | Y | Y | Y | P | Y | Y | N | P | Y | Y | Y | P | P | Y |  | P | 93% |
| 6.Study perspective | Y | N | Y | N | Y | N | N | P | Y | Y | P | N | N | Y | Y | Y | P | P | P | Y | Y | Y | N | Y | Y | P | Y | Y |  | Y | 76% |
| 7.Comparators | Y | Y | P | Y | Y | P | Y | Y | Y | Y | Y | Y | Y | P | NA | Y | Y | Y | Y | Y | Y | Y | P | NA | Y | Y | Y | Y |  | Y | 100% |
| 8.Time horizon | Y | P | Y | N | P | N | P | Y | Y | Y | P | Y | NA | Y | Y | Y | N | Y | P | Y | P | P | Y | Y | P | N | N | P |  | P | 82% |
| 9.Discount rate | N | N | NA | N | N | N | Y | P | N | Y | N | N | NA | Y | N | Y | N | P | NA | P | Y | N | NA | NA | P | P | P | N |  | NA | 48% |
| 10.Choice of health outcomes | NA | P | Y | P | Y | P | P | Y | NA | Y | NA | P | NA | Y | NA | Y | Y | NA | NA | P | P | P | NA | NA | P | Y | P | Y |  | Y | 100% |
| 11a.Measurement of effectiveness (single study-based estimates) | NA | NA | NA | N | Y | NA | NA | NA | NA | NA | NA | P | NA | P | NA | P | P | NA | NA | NA | NA | NA | NA | NA | P | P | P | P |  | NA | 90% |
| 11b.Measurement of effectiveness (synthesis-based estimates) | NA | NA | P | NA | NA | P | Y | Y | NA | Y | NA | P | NA | NA | NA | NA | NA | NA | NA | Y | Y | Y | NA | NA | NA | NA | NA | NA |  | Y | 100% |
| 12.Measurement and valuation of preference-based outcomes | NA | NA | NA | NA | NA | NA | N | NA | NA | NA | NA | N | NA | NA | NA | NA | NA | NA | NA | NA | Y | N | NA | NA | NA | NA | NA | NA |  | NA | 25% |
| 13a.Estimating resources and costs (single study-based economic evaluation) | Y | P | Y | NA | P | NA | N | N | Y | NA | Y | Y | Y | Y | Y | P | NA | Y | Y | NA | NA | NA | Y | Y | NA | NA | P | Y |  | Y | 90% |
| 13b.Estimating resources and costs (model-based economic evaluation) | NA | NA | NA | Y | NA | P | Y | Y | NA | Y | NA | P | NA | NA | NA | NA | Y | NA | NA | Y | N | Y | NA | NA | Y | P | NA | NA |  | NA | 92% |
| 14.Currency, price date, and conversion | Y | NA | Y | Y | Y | N | N | Y | Y | Y | P | P | N | P | Y | P | Y | Y | P | Y | N | Y | P | Y | N | P | P | Y |  | Y | 82% |
| 15.Choice of model | NA | NA | NA | N | NA | NA | P | P | NA | N | NA | Y | NA | NA | NA | NA | Y | NA | NA | P | Y | Y | NA | NA | P | N | N | NA |  | NA | 67% |
| 16.Assumptions | NA | NA | NA | N | NA | NA | Y | P | NA | P | NA | Y | NA | NA | NA | NA | Y | NA | NA | Y | P | NA | NA | NA | P | N | N | NA |  | NA | 73% |
| 17.Analytic methods | P | NA | NA | NA | NA | NA | NA | Y | Y | NA | NA | Y | NA | NA | NA | NA | Y | NA | NA | NA | NA | Y | NA | NA | NA | NA | P | NA |  | NA | 100% |
| **Results** | | | | | | | | | | | | | | | | | | | | | | | | | | | | | | | |
| 18.Study parameters | NA | P | Y | P | Y | Y | Y | Y | Y | Y | N | Y | N | Y | P | P | Y | Y | NA | Y | P | Y | Y | Y | Y | Y | P | Y |  | P | 93% |
| 19.Incremental costs and outcomes | Y | N | Y | Y | Y | Y | Y | Y | Y | Y | Y | Y | P | Y | Y | P | Y | Y | Y | Y | Y | Y | Y | Y | Y | Y | Y | P |  | Y | 97% |
| 20a.Characterizing uncertainty (single study-based economic evaluation) | P | N | P | NA | N | NA | NA | NA | P | NA | P | P | N | P | N | P | NA | P | N | NA | NA | NA | NA | Y | NA | NA | NA | N |  | N | 56% |
| 20b.Characterizing uncertainty (model-based economic evaluation) | NA | NA | NA | N | N | Y | P | Y | NA | P | NA | P | NA | NA | NA | NA | P | NA | NA | Y | Y | P | NA | NA | Y | P | P | NA |  | NA | 86% |
| 21.Characterizing heterogeneity | Y | P | NA | NA | NA | Y | NA | NA | Y | NA | NA | Y | NA | NA | NA | NA | NA | NA | NA | NA | NA | NA | NA | NA | NA | NA | Y | NA |  | Y | 100% |
| **Discussion** | | | | | | | | | | | | | | | | | | | | | | | | | | | | | | | |
| 22.Study findings, limitations, generalizability, and current knowledge | P | Y | P | Y | P | Y | Y | P | Y | Y | Y | P | Y | N | P | N | Y | Y | Y | Y | N | P | Y | Y | Y | Y | Y | Y |  | P | 90% |
| **Other** | | | | | | | | | | | | | | | | | | | | | | | | | | | | | | | |
| 23.Source of funding | N | P | P | P | N | N | P | Y | NA | Y | Y | N | P | Y | N | N | P | P | Y | P | N | P | N | Y | Y | N | N | N |  | N | 57% |
| 24.Conflicts of interest | N | N | N | N | N | Y | N | Y | NA | Y | Y | Y | N | Y | N | N | N | N | Y | Y | N | P | N | Y | Y | Y | N | N |  | N | 43% |
| **Reporting quality (% score for all 24 items*)** | Moderate (72%) | Moderate (53%) | Good (78%) | Low (48%) | Moderate (65%) | Moderate (58%) | Moderate (65%) | Good (83%) | Good (91%) | Good (88%) | Moderate (71%) | Moderate (61%) | Low (24%) | Good (79%) | Moderate (63%) | Moderate (63%) | Moderate (73%) | Good (76%) | Good (77%) | Good (90%) | Moderate (59%) | Moderate (73%) | Moderate (73%) | Good (97%) | Good (79%) | Moderate (62%) | Moderate (57%) | Moderate (71%) |  | Moderate (74%) | Low: 7% Moderate: 59% Good: 34% |
| **Reporting quality (% score excluding items 23 & 24)** | Good (81%) | Moderate (56%) | Good (84%) | Moderate (50%) | Moderate (72%) | Moderate (58%) | Moderate (69%) | Good (81%) | Good (91%) | Good (87%) | Moderate (67%) | Moderate (62%) | Low (24%) | Good (76%) | Moderate (71%) | Moderate (71%) | Good (78%) | Good (83%) | Moderate (73%) | Good (92%) | Moderate (65%) | Good (75%) | Good (85%) | Good (96%) | Good (76%) | Moderate (63%) | Moderate (62%) | Good (79%) |  | Good (82%) | Low: 3% Moderate: 45% Good: 52% |

Studies were assigned 2 points per item for Y, 1 point for P and 0 for N.

Abbreviations: Y: Fully considered. P: Partially considered. N: Not considered. NA: Not applicable.

**References**

1. Day K, Booyens S. The cost-effectiveness of managed care regarding chronic medicine prescriptions in a selected medical scheme. Curationis. 1998;21(4):65–70.

2. Edwards PR, Lunt DW, Fehrsen GS, Lombard CJ, Steyn K. Improving cost-effectiveness of hypertension management at a community health centre. S Afr Med J [Internet]. 1998 May;88(5):549–54. Available from: NL_INC

3. Anderson AN, Wessels F, Moodley I, Kropman K. AT1 receptor blockers--cost-effectiveness within the South African context. S Afr Med J [Internet]. 2000 May;90(5):494–8. Available from: NL_INC

4. Ker JA, Oosthuizen H, Rheeder P. Decision-making using absolute cardiovascular risk reduction and incremental cost-effectiveness ratios: A case study. Cardiovasc J Afr. 2008;19(2):97–101.

5. Makkink JL, Greeff OBW. Angiotensin converting enzyme inhibitors v. angiotensin receptor blockers in the management of hypertension: a funder’s perspective. S Afr Med J [Internet]. 2014 Apr;104(4):292–4. Available from: NL_EXC

6. Gaziano TA, Bertram M, Tollman SM, Hofman KJ. Hypertension education and adherence in South Africa: a cost-effectiveness analysis of community health workers. BMC Public Health [Internet]. 2014 Mar;14:240. Available from: NL_INC

7. Gaziano T, Abrahams-Gessel S, Surka S, Sy S, Pandya A, Denman CA, et al. Cardiovascular disease screening by community health workers can be cost-effective in low- resource countries. Health Aff [Internet]. 2015;34(9):1538–45. Available from: https://www.scopus.com/inward/record.uri?eid=2-s2.0-84942309984&doi=10.1377%2Fhlthaff.2015.0349&partnerID=40&md5=cc210ab65a3a2ef65981ffa0e53131c1

8. Basu S, Wagner RG, Sewpaul R, Reddy P, Davies J. Implications of scaling up cardiovascular disease treatment in South Africa: a microsimulation and cost-effectiveness analysis. Lancet Glob Heal [Internet]. 2019 Feb;7(2):e270–80. Available from: NL_INC

9. Nomame S. Development of a Model To Predict the Cost of Management of Diabetes Mellitus and Its Complications At Groote Schuur Hospital.

10. Volmink HC, Bertram MY, Jina R, Wade AN, Hofman KJ. Applying a private sector capitation model to the management of type 2 diabetes in the South African public sector: A cost-effectiveness analysis. BMC Health Serv Res [Internet]. 2014;14(1). Available from: https://www.scopus.com/inward/record.uri?eid=2-s2.0-84908086237&doi=10.1186%2F1472-6963-14-444&partnerID=40&md5=b35aa3f3e5075226b7573520ec168376

11. Erzse A, Stacey N, Chola L, Tugendhaft A, Freeman M, Hofman K. The direct medical cost of type 2 diabetes mellitus in South Africa: a cost of illness study. Glob Health Action. 2019;12(1):1–9.

12. Ncube-Zulu T, Danckwerts MP. Comparative hospitalization cost and length of stay between patients with and without diabetes in a large tertiary hospital in Johannesburg, South Africa. Int J Diabetes Dev Ctries. 2014;34(3):156–62.

13. Joannou J, Kalk WJ, Mahomed I, Ntsepo S, Berzin M, Joffe BI, et al. Screening for diabetic retinopathy in South Africa with 60° retinal colour photography. J Intern Med. 1996;239(1):43–7.

14. Khan T, Bertram MY, Jina R, Mash B, Levitt N, Hofman K. Preventing diabetes blindness: Cost effectiveness of a screening programme using digital non-mydriatic fundus photography for diabetic retinopathy in a primary health care setting in South Africa. Diabetes Res Clin Pract [Internet]. 2013;101(2):170–6. Available from: https://www.scopus.com/inward/record.uri?eid=2-s2.0-84881312079&doi=10.1016%2Fj.diabres.2013.05.006&partnerID=40&md5=0091ff0f25f877f5b2b785c4a63da005

15. Pepper DJ, Levitt NS, Cleary S, Burch VC. Hyperglycaemic emergency admissions to a secondary-level hospital - An unnecessary financial burden. South African Med J. 2007;97(10 I):963–7.

16. Wessels F. Is fenofibrate a cost-saving treatment for middle-aged individuals with type 2 diabetes? A South African private-sector perspective. Cardiovasc J Afr. 2010;21(1):43–6.

17. Gaziano T, Cho S, Sy S, Pandya A, Levitt NS, Steyn K. Increasing prescription length could cut cardiovascular disease burden and produce savings in south africa. Health Aff. 2015;34(9):1578–85.

18. Golovaty I, Sharma M, Van Heerden A, Van Rooyen H, Baeten JM, Celum C, et al. Cost of integrating noncommunicable disease screening into home-based HIV testing and counseling in South Africa. J Acquir Immune Defic Syndr. 2018;78(5):522–6.

19. Laäs DJ, Naidoo M. An evaluation of warfarin use at an urban district-level hospital in Kwazulu-natal Province, South Africa. South African Med J [Internet]. 2018;108(12):1046–50. Available from: https://www.scopus.com/inward/record.uri?eid=2-s2.0-85050767922&doi=10.7196%2FSAMJ.2018.v108i12.13256&partnerID=40&md5=d14555f575a953b532f3f7d6e9c32910

20. Lin JK, Moran AE, Bibbins-Domingo K, Falase B, Pedroza Tobias A, Mandke CN, et al. Cost-effectiveness of a fixed-dose combination pill for secondary prevention of cardiovascular disease in China, India, Mexico, Nigeria, and South Africa: a modelling study. Lancet Glob Heal. 2019;7(10):e1346–58.

21. Wessels F. Eprosartan in secondary prevention of stroke: The economic evidence. Cardiovasc J South Africa. 2007;18(2):95–6.

22. Bergh M, Marais CA, Miller-Jansön H, Salie F, Stander MP. Economic appraisal of dabigatran as first-line therapy for stroke prevention in atrial fibrillation. S Afr Med J [Internet]. 2013 Feb;103(4):241–5. Available from: nl_inc

23. Viljoen CA, Dalmeyer L, de Villiers L. Cost of acute stroke care in South Africa. Stroke. 2013;8(1):3–4.

24. Maredza M, Chola L. Economic burden of stroke in a rural South African setting. eNeurologicalSci [Internet]. 2016;3:26–32. Available from: https://www.scopus.com/inward/record.uri?eid=2-s2.0-84976633598&doi=10.1016%2Fj.ensci.2016.01.001&partnerID=40&md5=de78610df005a58dfa42b8561ca6fb64

25. Manyema M, Veerman LJ, Tugendhaft A, Labadarios D, Hofman KJ. Modelling the potential impact of a sugar-sweetened beverage tax on stroke mortality, costs and health-adjusted life years in South Africa. BMC Public Health [Internet]. 2016 May;16:405. Available from: nl_inc

26. Louw Q, Twizeyemariya A, Grimmer K, Leibbrandt D. Estimating the costs and benefits of stroke rehabilitation in South Africa. J Eval Clin Pract [Internet]. 2020;26(4):1181–7. Available from: https://doi.org/10.1111/jep.13287

27. Nixon Anderson A, Moodley I, Kropman K. A South African pharmaco-economic analysis of the acute infarction ramipril efficacy (AIRE) Study. Cardiovasc J South Africa. 2000;11(2):89–94.

28. Mabin TA, Condolfi P. An analysis of real-world cost-effectiveness of TAVI in South Africa. Cardiovasc J Afr [Internet]. 2014;25(1):21–6. Available from: nl_inc

29. Pestana JAX, Steyn K, Leiman A, Hartzenberg GM. The direct and indirect costs of cardiovascular disease in South Africa in 1991. South African Med J [Internet]. 1996;86(6):679–84. Available from: https://www.scopus.com/inward/record.uri?eid=2-s2.0-0029820322&partnerID=40&md5=9ccad6e5b25e6f0d00d46c3e590e1612

30. Biccard BM, Sear JW, Foëx P. The pharmaco-economics of peri-operative beta-blocker and statin therapy in South Africa. S Afr Med J [Internet]. 2006 Nov;96(11):1199–202. Available from: nl_inc
